# MAPK13 phosphorylates PHGDH and promotes its degradation via chaperone-mediated autophagy during liver injury

**DOI:** 10.1038/s41421-024-00758-w

**Published:** 2025-02-18

**Authors:** Ru Xing, Ruilong Liu, Yongxiao Man, Chen Liu, Yajuan Zhang, Hong Gao, Weiwei Yang

**Affiliations:** 1 Key Laboratory of Multi-cell Systems, Shanghai Key Laboratory of Molecular Andrology, CAS Center for Excellence in Molecular Cell Science, Shanghai Institute of Biochemistry and Cell Biology, University of Chinese Academy of Sciences, Chinese Academy of Sciences, Shanghai, China; 2 Ben May Department for Cancer Research, The University of Chicago, Chicago, IL USA; 3 Shanghai Institute of Thoracic Oncology, Shanghai Chest Hospital, Shanghai Jiao Tong University School of Medicine, Shanghai, China; 4 Key Laboratory of Systems Health Science of Zhejiang Province, School of Life Science, Hangzhou Institute for Advanced Study, University of Chinese Academy of Sciences, Hangzhou, Zhejiang, China

**Keywords:** Phosphorylation, Chaperone-mediated autophagy

## Abstract

Drug-induced liver injury (DILI) is the leading cause of acute liver failure and poses a significant clinical challenge in both diagnosis and treatment. Serine synthesis pathway (SSP) links glycolysis to one-carbon cycle and plays an important role in cell homeostasis by regulating substance synthesis, redox homeostasis and gene expression. However, the regulatory mechanism of SSP in DILI remains unclear. Phosphoglycerate dehydrogenase (PHGDH) is the rate-limiting enzyme in SSP. Here we show that during DILI, mitogen-activated protein kinase 13 (MAPK13) is activated and then phosphorylates PHGDH at serine 371 upon oxidative stress, which triggers PHGDH protein degradation via chaperone-mediated autophagy (CMA) pathway. PHGDH degradation suppresses SSP and glutathione production, thereby exacerbating DILI and cholestatic liver injury. Importantly, both MAPK13 inhibition and dietary serine supplementation ameliorates these liver injuries. Our finding demonstrates a unique regulatory mechanism of SSP, in which MAPK13 phosphorylates PHGDH and promotes its CMA degradation, establishes its critical role in DILI and cholestatic liver injury, and highlights the therapeutic potential of MAPK13 inhibitor or dietary serine to treat these liver injuries.

## Introduction

The liver functions as the primary organ responsible for drug metabolism, thereby making it a prime candidate for drug-induced injury (DILI). According to data provided by the World Health Organization (WHO), DILI has emerged as the fifth most common cause of liver disease-related deaths globally. The occurrence of DILI ranges between 1.4% and 8.1%, accounting for 20% to 50% of non-viral liver diseases and affecting ~ 20% of hospitalized patients with acute liver injury^1^. Notably, DILI is the leading cause of acute liver failure, comprising over half of all reported instances^2,3^. Many commonly prescribed drugs, including statins for high cholesterol, antibiotics, conventional chemotherapy drugs, and some new immune checkpoint inhibitor drugs like nivolumab and pembrolizumab, have been linked to liver toxicity cases^4–7^. Thus, studying the molecular and pathological mechanisms of DILI will enhance drug safety, aid in developing safer medications, and help develop biomarkers for early detection and therapeutic targets for this severe drug reaction.

The liver injury process involves the reprogramming of cellular metabolism. During acute-on-chronic liver failure (ACLF), the activities of glycolysis and pentose phosphate pathway are increased, while mitochondrial oxidative phosphorylation and fatty acid β-oxidation are decreased^8^. In mice with hepatic steatosis, reversed serine hydroxymethyltransferase 2 (SHMT2) activity drives glycine depletion and acetaminophen hepatotoxicity^9^. Reduced expression of phosphoglycerate dehydrogenase (PHGDH), the rate-limiting enzyme in the serine synthesis pathway (SSP) and serum serine levels are closely associated with the development of fatty liver disease^10^. Some clinical data also implicate that serum serine levels are lower in patients with ACLF than in healthy individuals^11^. The levels of PHGDH protein in serum are negatively correlated with the progression of fibrosis in patients with alcoholic liver disease^12^. These studies provide a hint about the possible role of SSP, PHGDH and serine in various forms of liver injuries.

Oxidative stress serves as the primary stress responsible for a variety of clinical liver injuries, such as liver ischemia/reperfusion (I/R) during liver surgery^13^, chronic liver inflammation caused by alcohol or cholestasis^14,15^, or liver injury caused by a variety of drugs such as chemotherapy and antipyretic drugs^16,17^. SSP is the link between glycolysis and one-carbon cycle and plays an important role in cell homeostasis by contributing to substance synthesis, epigenetic regulation and redox homeostasis regulation in cells^18–23^. In the current study, we investigated the role and regulatory mechanism of SSP in DILI and found that during the injury process, oxidative stress-induced MAPK13-regulated PHGDH protein degradation attenuates SSP flux and glutathione (GSH) production, thereby inhibiting the survival of hepatocytes and aggravating liver injury. MAPK13 inhibition and dietary serine supplementation can alleviate both acute and chronic liver injuries.

## Results

### Hepatic PHGDH knockout exacerbates liver injury

**Fig. 1:**
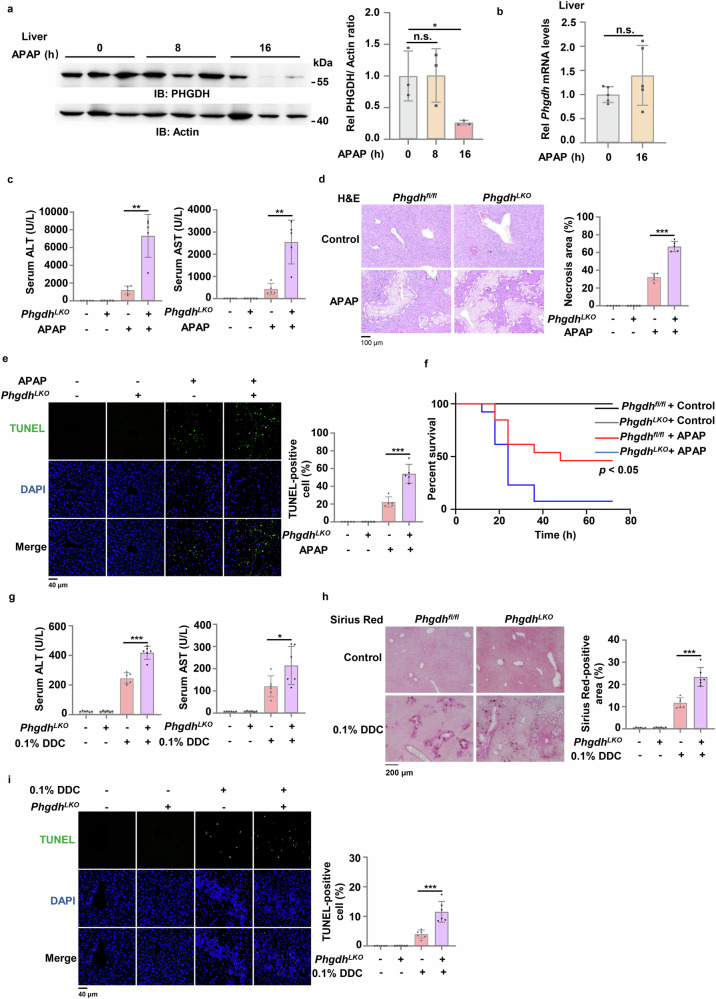
Hepatic PHGDH deficiency exacerbates oxidative stress-associated acute or chronic liver injury. **a** c57BL/6J mice were intraperitoneally injected with 250 mg/kg APAP. Livers were collected at 0 h, 8 h or 16 h after APAP treatment (*n* = 3 in each time point). Immunoblotting analysis was performed and shown on the left panel. The statistical analysis of relative protein levels was shown on the right panel. IB, immunoblotting. **b** c57BL/6 J mice were intraperitoneally injected with 250 mg/kg APAP. Livers were collected at 0 h or 16 h after APAP treatment. *Phgdh* mRNA levels in mouse livers were examined (*n* = 5 in each time point). **c**‒**e** *Phgdh*^*fl/fl*^ and *Phgdh*^*LKO*^ mice were intraperitoneally injected with normal saline (control) or 250 mg/kg APAP. 24 h after treatment, serum ALT and AST levels were detected by kit (**c**). Liver necrosis area in H&E-stained sections were circled and quantified (**d**). Positive liver cells in TUNEL-stained sections were quantified (**e**). Representative images of H&E staining or TUNEL staining were shown on the left panel and statistical analysis were shown on the right panel (*n* = 5 per group). **f** Survival of *Phgdh*^*fl/fl*^ and *Phgdh*^*LKO*^ mice intraperitoneally injected with normal saline (control) or 750 mg/kg APAP during 72 h (*n* = 13 per group). **g**‒**i** *Phgdh*^*fl/fl*^ and *Phgdh*^*LKO*^ mice were fed with irradiated diet with or without 0.1% DDC for 2 weeks. Serum ALT and AST levels were detected in these mice (**g**). Mouse livers were dissected and subjected to Sirius Red staining (**h**) and TUNEL staining (**i**) (*n* = 6 per group). Survival data are based on Long-rank test (**f**). Other data are means ± SD. Each point represents an individual mouse. *P* values were determined by two-tailed Student’s *t*-test.

Acetaminophen (APAP) overdose is the most commonly used treatment for generating clinically relevant model of DILI. Mechanistically, the toxicity is initiated by conversion of APAP to an electrophile thought to be N-acetyl-p-benzoquinone imine (NAPQI). NAPQI binds to sulfhydryl groups on glutathione, which leads to the depletion of GSH and sensitizes the cells to oxidative stress^24^. To investigate how metabolism is regulated during DILI, we performed a metabolomic analysis in liver tissues from APAP-injured mice, showing that the serine, glycine and threonine metabolism was enriched after APAP treatment (Supplementary Fig. S1a). To investigate the role of SSP in DILI, we adopted APAP model and examined the expression of PHGDH. The protein levels of PHGDH were gradually decreased in liver tissues dissected from APAP-treated mice (Fig. 1a; Supplementary Fig. S1b), while its mRNA levels were not influenced by APAP treatment (Fig. 1b). Hepatocytes are the most abundant and functional cell population, accounting for more than 80% cells in the liver and also the major cell population that contributes to the liver regeneration^25^. We then examined PHGDH expression in APAP-treated primary hepatocytes. Similar results were obtained, showing that the levels of PHGDH protein, but not its mRNA levels, were similarly decreased in APAP-treated cells (Supplementary Fig. S1c, d), suggesting the potential role of reprogrammed SSP in APAP-induced acute liver injury.

To test the hypothesis, we first examined the expression pattern of PHGDH in mouse liver tissues, showing that PHGDH was expressed in hepatocytes and higher expression levels of PHGDH were detected in hepatocytes located near the central and peripheral veins (Supplementary Fig. S1e). We thus generated the hepatocyte-specific PHGDH knockout mice by crossing *Phgdh*^*flox/flox*^ (*Phgdh*^*fl/fl*^) mice with Alb-cre mice, the transgenic mice expressing Cre recombinase under the control of the mouse albumin promoter (Supplementary Fig. S1f). The efficiency of hepatic *Phgdh* knockout was validated by examining the mRNA and protein expression of PHGDH in liver tissues and hepatocytes (Supplementary Fig. S1g‒i). Of note, *Phgdh* knockout in hepatocytes did not result in obvious liver injury and the loss of liver weight in mice (Supplementary Fig. S1j‒l). We next treated *Phgdh* wild-type (WT) mice (*Phgdh*^*fl/fl*^) and hepatic *Phgdh* knockout mice (*Phgdh*^*LKO*^) with APAP and examined hepatoxicity in these mice. *Phgdh*^*LKO*^ mice exhibited much higher levels of alanine aminotransferase (ALT) and aspartate aminotransferase (AST) in serum after APAP treatment than *Phgdh* WT mice (Fig. 1c). Hematoxylin and eosin (H&E) staining showed that *Phgdh* knockout increased APAP-induced necrosis in hepatic lobule (Fig. 1d). At cellular level, more dead cells were observed in PHGDH knockout mice after APAP treatment than in *Phgdh* WT mice, as determined by TUNEL assay (Fig. 1e). Consistently, the survival rate of the mice treated with the lethal dose of APAP was markedly reduced by *Phgdh* knockout (Fig. 1f). Taken together, these results demonstrate a protective role of PHGDH in acute liver injury.

In addition, we also determined the role of PHGDH in chronic liver injury by adopting the mouse model of 3,5-diethoxycarbonyl-1,4-dihydrocollidine (DDC)-induced cholestatic liver injury^15^. Many studies have shown that cholestatic liver injury is a neutrophil-mediated inflammatory response, and oxidative stress induced by neutrophils is the main mechanism of hepatocytes death^26^. After two weeks of DDC diet, we observed that *Phgdh*^*LKO*^ mice exhibited much higher levels of ALT and AST in serum than *Phgdh*^*fl/fl*^ mice (Fig. 1g). H&E staining showed spotty necrosis of hepatocytes was aggravated in *Phgdh*^*LKO*^ mice (Supplementary Fig. S1m). Sirius Red staining indicated that PHGDH knockout greatly enhanced DDC-promoted liver fibrosis (Fig. 1h). And more dead cells were observed in *Phgdh*^*LKO*^ mice than in *Phgdh*^*fl/fl*^ mice (Fig. 1i). These results confirm the protective role of PHGDH in chronic liver injury.

### PHGDH is degraded via CMA pathway in hepatocytes upon oxidative stress

**Fig. 2:**
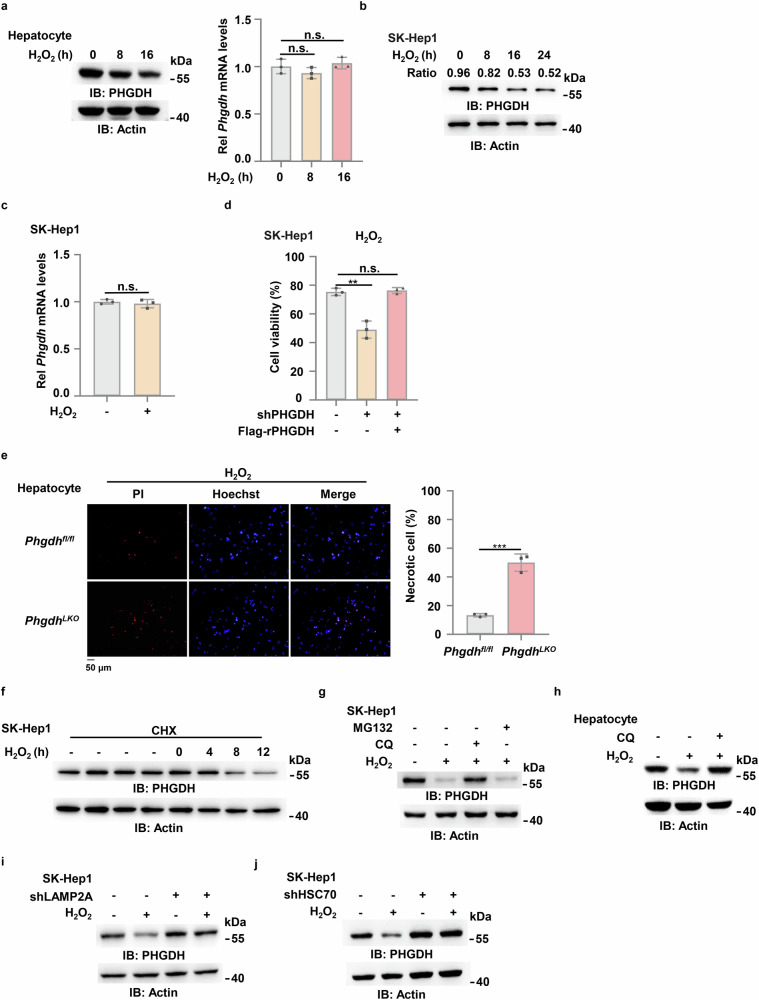
PHGDH is degraded via CMA pathway in hepatocytes upon oxidative stress. **a** Primary hepatocytes from c57BL/6 J mice were treated with 0.5 mM H_2_O_2_ for 0 h, 8 h or 16 h. PHGDH protein (left) and mRNA (right) levels were examined. IB, immunoblotting. **b, c** SK-Hep1 cells were treated with 0.5 mM H_2_O_2_ for 0 h, 8 h, 16 h or 24 h. Immunoblotting analysis was performed and the ratio of gray values of PHGDH to Actin were shown (**b**). The mRNA (right) levels at 16 h were examined (**c**). **d** SK-Hep1 cells stably expressing shNT or shPHGDH were reconstituted with or without Flag-rPHGDH and then treated with 0.5 mM H_2_O_2_ for 24 h. Cell viability was determined using trypan blue staining. **e** Primary hepatocytes isolated from *Phgdh*^*fl/fl*^ and *Phgdh*^*LKO*^ mice were treated with 0.5 mM H_2_O_2_ for 24 h. Cells were stained with 1 μg/mL propidium iodide (PI) and 10 μg/mL Hoechst, and the percentage of dead cells was quantified. **f** SK-Hep1 cells were treated with 50 μg/mL CHX and treated with or without 0.5 mM H_2_O_2_ as indicated time. **g** SK-Hep1 cells were treated with 0.5 mM H_2_O_2_ and 20 μM Chloroquine (CQ) or 10 μM MG132 for 24 h. **h** Hepatocytes from c57BL/6 J mice were treated with 0.5 mM H_2_O_2_ and 10 μM CQ for 16 h. **i** SK-Hep1 cells stably expressing shNT or shLAMP2A were treated with 0.5 mM H_2_O_2_ for 24 h. **j** SK-Hep1 cells stably expressing shNT or shHSC70 were treated with 0.5 mM H_2_O_2_ for 24 h. Immunoblots are representative of three independent experiments. Data represent the means ± SD of three independent experiments. *P* values were determined by two-tailed Student’s *t*-test.

As we demonstrated earlier, the protein levels of PHGDH, but not its mRNA levels, were downregulated in liver tissues or primary hepatocytes after APAP treatment (Fig. 1a, b; Supplementary Fig. S1c, d). Next, we explored the regulatory mechanism of PHGDH expression during liver injury. The toxicity of APAP is mainly fulfilled by depleting intracellular GSH, which exposes hepatocytes to oxidative stress. To simulate this scenario, we treated the primary mouse hepatocytes or human hepatic cell carcinoma cell line, SK-Hep1, with hydrogen peroxide (H_2_O_2_) and observed a similar result in APAP-treated mouse model, showing that the levels of PHGDH protein, but not its mRNA levels, were gradually decreased in the cells after H_2_O_2_ treatment (Fig. 2a‒c). Moreover, we depleted *PHGDH* in SK-Hep1 cells with shRNA specifically against *PHGDH* and observed that *PHGDH* depletion dramatically decreased the viability of the cells after H_2_O_2_ treatment, while rescued expression of shRNA-resistant PHGDH (rPHGDH) completely recovered the viability of *PHGDH*-depleted cells, excluding the off-targeting possibility of *PHGDH* shRNA (Fig. 2d; Supplementary Fig. S2a). Similar results were obtained in the experiments with the primary hepatocytes with or without *PHGDH* knockout (Fig. 2e). Consistent with the results in liver-injured mice, these results indicate that oxidative stress induces the decrease of PHGDH protein in hepatocytes, which sensitizes the cells to oxidative stress.

To investigate how oxidative stress induces PHGDH downregulation, we examined the degradation of PHGDH protein by treating SK-Hep1 cells with cycloheximide (CHX), a translation elongation inhibitor (Supplementary Fig. S2b). The degradation of PHGDH protein was greatly accelerated in the cells upon H_2_O_2_ treatment (Fig. 2f). Controlled protein degradation plays an important role in maintaining cellular homeostasis and its dysregulation is closely related to various pathological process. The principal sites for protein degradation in cells are lysosomes and proteasomes. Both are involved in the constitutive turnover of cellular proteins. Typically, short-lived proteins are degraded by proteasomes, whereas lysosomes are responsible for the degradation of long-lived proteins^27^. To determine by which pathway PHGDH protein is degraded, we incubated SK-Hep1 cells with the proteasome inhibitor (MG132) or lysosome inhibitor (Qunoline, CQ). The treatment of CQ, but not that of MG132, blocked H_2_O_2_-induced PHGDH degradation (Fig. 2g). Similar results were obtained in the experiment with primary hepatocytes (Fig. 2h), suggesting that the lysosomal pathway is responsible for PHGDH protein degradation in hepatocytes upon oxidative stress.

Chaperone-mediated autophagy (CMA) was the first discovered lysosomal process by which intracellular components are selectively degraded. CMA involves the selective degradation of KFERQ-like motif-bearing proteins delivered to the lysosomes via chaperone HSC70 and cochaperones, such as carboxyl terminus of HSC70-interacting protein (CHIP), heat shock protein 40 (DNABJ1) and HSP70-HSP90 organizing protein (HOP), and their internalization in lysosomes via the receptor lysosome-associated membrane protein type 2A (LAMP2A)^28^. To test whether PHGDH is degraded via CMA pathway, we depleted two key components of the pathway, including lysosome-associated membrane protein type 2A (LAMP2A) and heat shock cognate 71 kDa protein (HSC70) (Supplementary Fig. S2c, d) in SK-Hep1 cells. Depletion of either LAMP2A or HSC70 completely recovered the levels of PHGDH protein in the cells after H_2_O_2_ treatment (Fig. 2i, j).

Collectively, these results demonstrate that PHGDH is degraded via CMA pathway in hepatocytes upon oxidative stress.

### S371 phosphorylation triggers CMA degradation of PHGDH protein

**Fig. 3:**
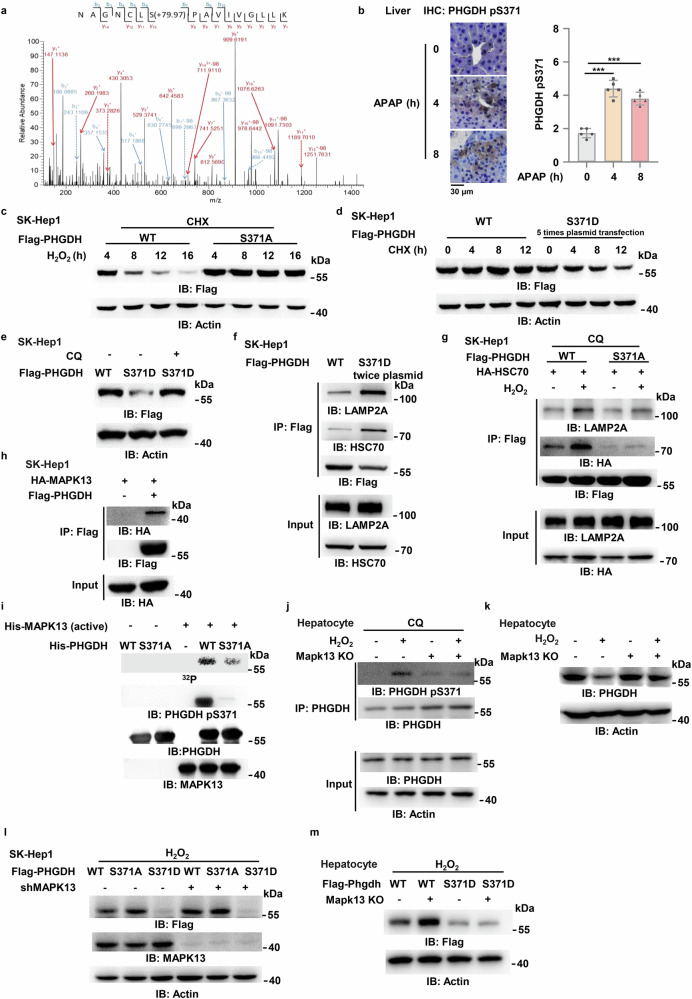
MAPK13 phosphorylates PHGDH at S371 and promotes its protein degradation upon oxidative stress. **a** SFB-PHGDH was pulled down from SK-Hep1 cells stably expressing SFB-PHGDH treated with or without 0.5 mM H_2_O_2_ and 20 μM CQ for 3 h. The mass spectra of peptide harboring modified S371 residue was presented. Predicted b-type and y-type ions (not including all) were listed above and below the peptide sequence, respectively. **b** c57BL/6 J mice were intraperitoneally injected with 250 mg/kg APAP. Mouse livers were collected at 0 h, 4 h or 8 h after APAP treatment. Immunohistochemistry (IHC) staining of liver section using anti-PHGDH pS371 antibody was performed. Representative images of IHC staining were presented on the left panel, and statistical analysis were shown on the right panel (*n* = 5 in each time point). **c** SK-Hep1 cells were transiently transfected with Flag-PHGDH or Flag-PHGDH S371A. Cells were then treated with 0.5 mM H_2_O_2_ and 50 μg/mL CHX as indicated time. IB, immunoblotting. **d** SK-Hep1 cells were transiently transfected with Flag-PHGDH WT or 5 times Flag-PHGDH S371D plasmid and then treated with 50 μg/mL CHX as indicated time. **e** SK-Hep1 cells were transiently transfected with Flag-PHGDH WT or Flag-PHGDH S371D and then treated with or without 20 μM CQ for 16 h. **f** Flag-PHGDH was immunoprecipitated from SK-Hep1 cells transiently transfected with Flag-PHGDH WT or twice dose of Flag-PHGDH S371D plasmid. 36 h after transfection, the endogenous interacting proteins (LAMP2A and HSC70) were detected. **g** SK-Hep1 cells were transiently transfected with Flag-PHGDH WT or Flag-PHGDH S371A and HA-HSC70. 24 h after transfection, the cells were treated with 20 μM CQ and Flag-PHGDH was immunoprecipitated from the cells after treated with or without 0.5 mM H_2_O_2_ for 12 h. The interacting proteins (LAMP2A and HA-HSC70) were detected. **h** Flag-PHGDH was immunoprecipitated from SK-Hep1 cells co-transfected with EV or Flag-PHGDH and HA-MAPK13. **i** In vitro kinase assays were performed by mixing purified His-MAPK13 F324S and His-PHGDH WT or His-PHGDH S371A protein. **j** PHGDH was immunoprecipitated from hepatocytes isolated from WT and *Mapk13* KO mice which were then treated with or without 0.5 mM H_2_O_2_ and 10 μM CQ for 4 h. **k** Hepatocytes isolated from WT and *Mapk13* KO mice were treated with or without 0.5 mM H_2_O_2_ for 16 h. **l** SK-Hep1 cells stably expressing shNT or shMAPK13 were transiently transfected with Flag-PHGDH, Flag-PHGDH S371A or Flag-PHGDH S371D, and then treated with 0.5 mM H_2_O_2_ for 24 h. **m** Hepatocytes isolated from WT and *Mapk13* KO mice transiently transfected with Flag-Phgdh WT or Flag-Phgdh S371D. Cells were treated with 0.5 mM H_2_O_2_ for 16 h. Immunoblots are representative of three independent experiments. Data are means ± SD. Each point represents an individual mouse. *P* values were determined by two-tailed Student’s *t*-test.

Post-translational modification (PTM), often occurring on specific amino acid residue within regulatory domain of target protein, controls the stability of the protein. Phosphorylation is one of the most abundant functional forms of PTM. To explore how CMA degradation of PHGDH protein is triggered upon oxidative stress, we performed mass spectrometry analysis of PHGDH phosphorylation in H_2_O_2_-treated SK-Hep1 cells. Among the two phosphorylated residues, the level of PHGDH serine (S) 371 phosphorylation (PHGDH pS371) was most dramatically enhanced in the cells after H_2_O_2_ treatment (Fig. 3a; Supplementary Fig. S3a). Of note, ubiquitinated proteins can also be degraded via the lysosomal pathway^29^. We thus examined the ubiquitination of PHGDH, which showed that no significant changes in PHGDH ubiquitination were observed in the cells after H_2_O_2_ treatment (Supplementary Fig. S3b).

To confirm that PHGDH pS371 indeed occurs in hepatocytes or liver tissues, we generated a custom-designed antibody specifically against phosphorylated PHGDH S371. Firstly, as shown in Supplementary Fig. S3c, this antibody could recognize PHGDH WT and phospho-mimic PHGDH S371D mutant, in which S371 was mutated into aspartic acid (D), but failed to recognize phospho-deficient PHGDH S371A mutant, in which S371 was mutated into alanine (A), in SK-Hep1 cells after H_2_O_2_ treatment, confirming the specificity of anti-PHGDH pS371 antibody. Secondly, by using this antibody, we observed that PHGDH pS371 was markedly induced in SK-Hep1 cells after H_2_O_2_ treatment (Supplementary Fig. S3d). Moreover, immunohistochemistry staining with the antibody in dissected liver tissues from APAP-treated mice showed that PHGDH pS371 was markedly increased after APAP treatment (Fig. 3b).

Next, we determined whether PHGDH pS371 influences its protein stability. As shown in Fig. 3c, S371A mutation markedly enhanced the stability of PHGDH protein in SK-Hep1 cells after H_2_O_2_ treatment. In contrast, S371D mutation decreased the stability of PHGDH protein even in the absence of H_2_O_2_ treatment (Fig. 3d). To confirm whether pS371 induces PHGDH degradation via CMA, we transfected SK-Hep1 cells with Flag-PHGDH WT or S371D and treated the cells with or without CQ. The treatment with CQ successfully restored the levels of PHGDH S371D protein to those comparable with PHGDH WT protein (Fig. 3e). Similarly, the levels of PHGDH S371D protein were also restored by HSC70 depletion (Supplementary Fig. S3e). In addition, compared to PHGDH WT, PHGDH S371D showed stronger interaction with LAMP2A and HSC70, the key components of CMA pathway (Fig. 3f). However, S371A mutation abrogated H_2_O_2_-induced interaction with LAMP2A and HSC70 (Fig. 3g). Taken together, these results demonstrate that PHGDH pS371 is induced and required for CMA degradation of PHGDH in hepatocytes upon oxidative stress.

### MAPK13 phosphorylates PHGDH at S371

To identify the upstream kinase responsible for PHGDH pS371, we conducted a kinase prediction program according to the amino acid residues surrounding S371 by using a kinase-library website (https://kinase-library.phosphosite.org), integrated with the analysis of PHGDH-interacted proteins with HitPredict database (http://www.hitpredict.org). By using this strategy, we identified MAPK13 as the potential kinase to phosphorylate PHGDH S371 (Supplementary Fig. S3f, g). MAPK13, also known as the p38δ, is one of the subtypes of the p38 MAPK family and is a less-studied subtype of the p38 MAPK family. As one of the important subfamilies of the MAPK pathway, this family is closely related to inflammation, apoptosis and growth. As a serine/threonine kinase, it has certain selectivity for substrate proteins and its substrate proteins usually contain XX(S/T)PX motif^30–32^. We noticed that the amino acid residues surrounding S371 were similar to the consensus motif of MAPK substrate and conserved among multiple species (Supplementary Fig. S3h).

To confirm whether MAPK13 phosphorylates PHGDH S371, we first performed co-immunoprecipitation (co-IP) assay, showing that MAPK13 interacted with PHGDH (Fig. 3h). Moreover, we expressed and purified recombinant His-PHGDH WT or S371A and active His-MAPK13 in bacteria and conducted in vitro kinase assay with these recombinant proteins. MAPK13 effectively phosphorylated PHGDH WT; however, the phosphorylation level in the PHGDH S371A group was significantly reduced (Fig. 3i). Meanwhile, we performed mass spectrometry analysis of PHGDH phosphorylation after the in vitro kinase assay, which showed that multiple residues on PHGDH, including S341, S371, S14, S251, T147 and T353, were phosphorylated by MPAK13 and S371 ranked top 2 among the phosphorylated residues (Supplementary Fig. S3i). Moreover, MAPK13 depletion abrogated H_2_O_2_ treatment-induced PHGDH pS371 in primary hepatocytes and SK-Hep1 cells (Fig. 3j; Supplementary Fig. S3j, k).

To determine whether MAPK13 promotes PHGDH degradation by phosphorylating S371, we depleted MAPK13 in SK-Hep1 cells and observed that MAPK13 depletion abrogated H_2_O_2_-induced PHGDH degradation (Supplementary Fig. S3l). Similar results were obtained in primary hepatocytes dissected from *Mapk13* knockout mice (Fig. 3k). Additionally, unlike PHGDH WT, PHGDH S371A or S371D degradation was not influenced by MAPK13 depletion in SK-Hep1 cells and hepatocytes after H_2_O_2_ treatment (Fig. 3l, m). Collectively, these results demonstrate that MAPK13 phosphorylates PHGDH S371 in hepatocytes upon oxidative stress, thereby promoting PHGDH degradation.

### MAPK13-dependent PHGDH pS371 sensitizes hepatocytes to oxidative stress-induced cell death

**Fig. 4:**
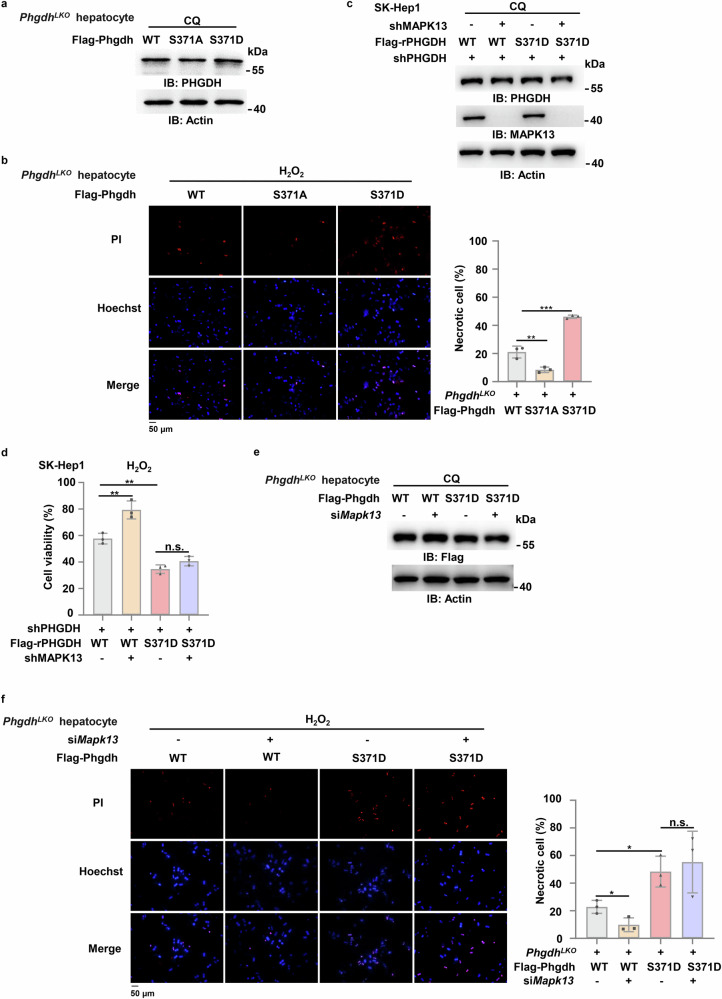
MAPK13-dependent PHGDH pS371 sensitizes hepatocytes to oxidative stress-induced cell death. **a**, **b** Primary hepatocytes isolated from *Phgdh*^*LKO*^ mice were transfected with Flag-Phgdh WT, Flag-Phgdh S371A or Flag-Phgdh S371D. Cells were treated with 10 μM CQ for 24 h (**a**). Transfected cells were treated with 0.5 mM H_2_O_2_ for 24 h. Cells were stained with 1 μg/mL PI and 10 μg/mL Hoechst, and the percentage of dead cells was quantified (**b**). **c**, **d** SK-Hep1 cells stably expressing shPHGDH were infected with lentivirus expressing shNT or shMAPK13 and then infected with lentivirus expressing Flag-rPHGDH WT or Flag-rPHGDH S371D respectively. Cells then were treated with 20 μM CQ for 24 h (**c**). Infected cells were treated with 0.5 mM H_2_O_2_ for 24 h. Cell viability was determined using trypan blue staining (**d**). **e**, **f** Primary hepatocytes isolated from *Phgdh*^*LKO*^ mice were transfected with siNC or si*Mapk13* and then transfected with Flag-Phgdh WT or Flag-Phgdh S371D. Cells were treated with 10 μM CQ for 24 h (**e**). Transfected cells were treated with 0.5 mM H_2_O_2_ for 24 h. Cells were then stained with 1 μg/mL PI and 10 μg/mL Hoechst, and the percentage of dead cells was quantified (**f**). Immunoblots are representative of three independent experiments. Data represent the means ± SD of three independent experiments. *P* values were determined by two-tailed Student’s *t*-test.

To investigate the role of PHGDH pS371 in the survival of hepatocytes under oxidative stress, we depleted PHGDH in SK-Hep1 cells and rescued the cells with rPHGDH WT, S371A or S371D (Supplementary Fig. S4a). Compared to the cells expressing rPHGDH WT, the cells expressing rPHGDH S371A exhibited higher cell viability, while more dead cells were observed in the cells expressing rPHGDH S371D after H_2_O_2_ treatment (Supplementary Fig. S4b). Similar results were obtained in *Phgdh*^*LKO*^ hepatocytes rescued with PHGDH WT, S371A or S371D (Fig. 4a), showing that compared to that of PHGDH WT, the expression of PHGDH S371A increased the viability of hepatocytes, while the expression of PHGDH S371D decreased the viability of the cells after H_2_O_2_ treatment (Fig. 4b). *N*-acetylcysteine (NAC) is a potent antioxidant derived from the amino acid L-cysteine. We next treated PHGDH-depleted SK-Hep1 cells expressing rPHGDH WT and rPHGDH S371D with or without NAC. The viability of the cells expressing rPHGDH S371D was restored by NAC treatment to that of the cells expressing rPHGDH WT after H_2_O_2_ treatment (Supplementary Fig. S4c), suggesting that PHGDH pS371 aggravates oxidative stress-induced cell death by attenuating antioxidant capacity. Collectively, these results indicate that PHGDH pS371 is detrimental for hepatocytes’ viability under oxidative stress.

To confirm that MAPK13 promotes oxidative stress-induced hepatocyte death by phosphorylating PHGDH S371, we depleted MAPK13 in PHGDH-depleted SK-Hep1 cells rescued with rPHGDH WT or S371D cells (Fig. 4c). MAPK13 depletion recovered the viability of the cells expressing rPHGDH WT, but failed to recover that of the cells expressing rPHGDH S371D after H_2_O_2_ treatment (Fig. 4d). Similar result was obtained in primary hepatocytes (Fig. 4e, f; Supplementary Fig. S4d). Collectively, these results demonstrate that MAPK13 promotes oxidative stress-induced hepatocyte death by phosphorylating S371.

### MAPK13 inhibition alleviates acute or chronic liver injury

**Fig. 5:**
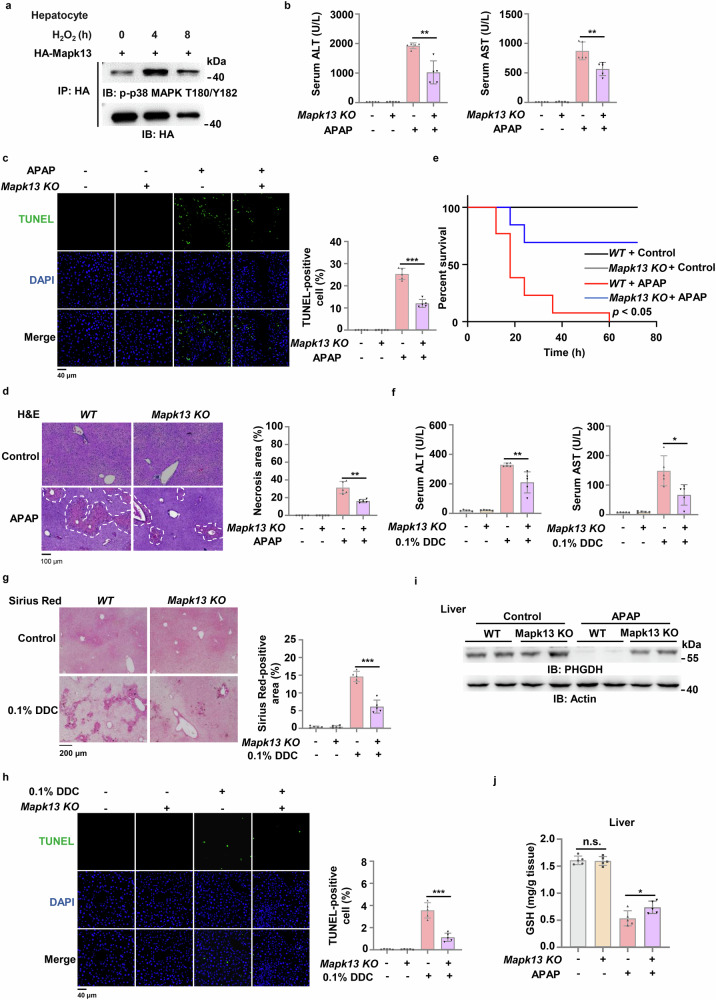
MAPK13 deficiency alleviates oxidative stress-associated acute or chronic liver injury. **a** Primary hepatocytes isolated from c57BL/6 J mice were transiently transfected with HA-Mapk13. Cells were then treated with 0.5 mM H_2_O_2_ for 0 h, 4 h or 8 h. HA-Mapk13 was immunoprecipitated and phosphorylation of Mapk13 was detected. **b**‒**d** WT and *Mapk13* KO mice were intraperitoneally injected with normal saline (control) or 300 mg/kg APAP. After 24 h treatment, serum ALT and AST levels in these mice were detected by kit (**b**). Positive liver cells in TUNEL-stained sections were quantified (**c**). Liver necrosis area in H&E-stained sections were circled and quantified (**d**). Representative images of H&E staining or TUNEL staining were shown on the left panel and statistical analysis were shown on the right panel (*n* = 5 per group). **e** Survival during 72 h of WT and *Mapk13* KO mice intraperitoneally injected with normal saline (control) or 800 mg/kg APAP (*n* = 13 per group). **f**‒**h** WT and *Mapk13* KO mice were fed with irradiated diet with or without 0.1% DDC for 2 weeks. Serum ALT and AST levels were detected in these mice (**f**). Mouse livers were dissected and subjected to Sirius Red staining (**g**) and TUNEL staining (**h**) (*n* = 5 per group). **i** WT and *Mapk13* KO mice were intraperitoneally injected with normal saline (control) or 300 mg/kg APAP. Livers were collected at 16 h after treatment. Immunoblotting analysis was performed (*n* = 2 per group). **j** WT and *Mapk13* KO mice were intraperitoneally injected with normal saline or 300 mg/kg APAP. The GSH level in mouse livers was measured (*n* = 5 per group). Immunoblots are representative of three independent experiments. Survival data are based on Long-rank test (**e**). Other data are means ± SD. Each point or lane represents an individual mouse. *P* values were determined by two-tailed Student’s *t*-test.

To confirm the role of MAPK13 in liver injury, we first examined whether MAPK13 is activated in hepatocytes upon oxidative stress. We observed that MAPK13 was activated in primary hepatocytes after H_2_O_2_ treatment, as indicated by the phosphorylation of p38 MAPK T180/Y182 (Fig. 5a). Of note, the mRNA levels of MAPK13 did not change in mouse liver tissues after APAP treatment or in hepatocytes with H_2_O_2_ treatment (Supplementary Fig. S5a, b). MKK3/6 are the classical kinases that mediate the activation of the p38 MAPKs. To determine whether oxidative stress activates MAPK13 via MKK3/6, we treated mouse hepatocytes with Gossypetin, an MKK3/6 inhibitor. Gossypetin treatment suppressed MAPK13 activation induced by oxidative stress (Supplementary Fig. S5c), suggesting that MKK3/6 is required for oxidative stress-induced MAPK13 activation. We next treated *Mapk13* knockout mice with APAP. As shown in Fig. 5b‒d, the levels of ALT and AST in serum were decreased, the numbers of dead cells were reduced in liver tissues and less necrosis was detected in hepatic lobule in APAP-treated mice. Moreover, *Mapk13* knockout dramatically prolonged the survival duration of the mice treated with lethal dose of APAP (Fig. 5e). Similarly, we observed that *Mapk13* knockout mice exhibited much lower levels of ALT and AST in serum (Fig. 5f), less liver fibrosis area (Fig. 5g) and fewer dead cells (Fig. 5h) in liver tissues in DDC-treated mice. These results demonstrate that MAPK13 activation sensitizes mice to APAP or DDC-induced liver injury, suggesting that MAPK13 inhibition can alleviate both acute and chronic liver injury.

In addition, we harvested liver tissues from the mice treated with APAP. MAPK13 knockout increased the protein levels of PHGDH, but not the mRNA levels of PHGDH (Fig. 5i; Supplementary Fig. S5d). And higher levels of GSH were detected in liver tissues dissected from *Mapk13* knockout mice than those in *Mapk13* WT mice (Fig. 5j).

NAC is currently the only clinically used drug to alleviate APAP-induced hepatotoxicity, but it can exert maximum effect only when administered within 8 h of poisoning. To further evaluate the therapeutic potential of MAPK13 inhibitor for DILI, we administrated the mice with MAPK13 inhibitor (MAPK13-IN-1) 10 h after APAP treatment. We observed that MAPK13-IN-1 administration decreased serum ALT and AST levels, reduced the number of dead cells in liver tissues, and inhibited necrosis in hepatic lobule of the mice treated with APAP (Supplementary Fig. S5e‒g). In addition, we observed that MAPK13-IN-1 increased PHGDH protein levels in livers (Supplementary Fig. S5h). These results indicate that MAPK13-IN-1 can alleviate DILI.

### MAPK13-dependent PHGDH pS371 reduces SSP flux and GSH production

**Fig. 6:**
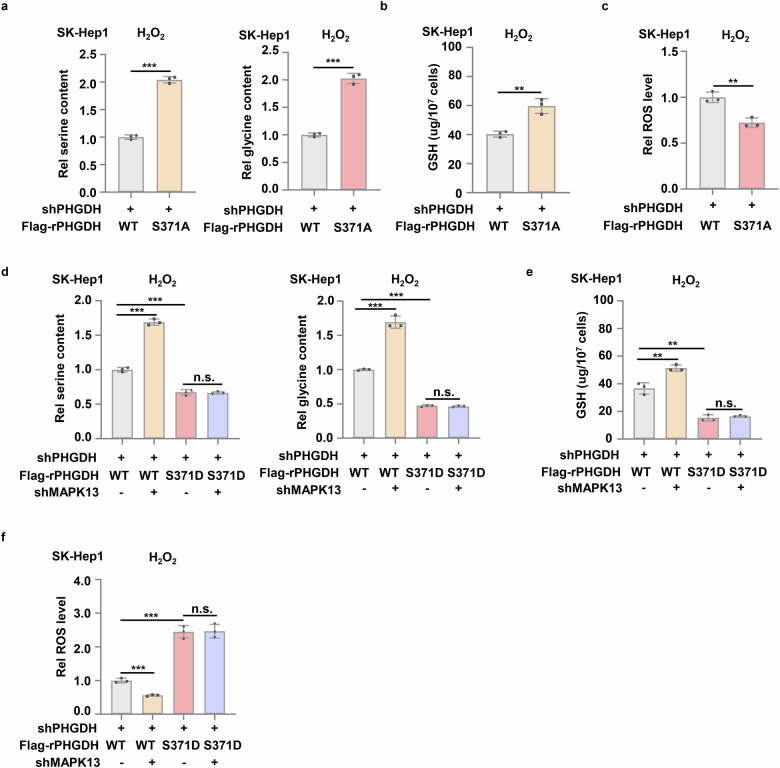
MAPK13-dependent PHGDH pS371 reduces SSP flux and GSH production. **a**‒**c** SK-Hep1 cells stably expressing shPHGDH were infected with lentivirus expressing Flag-PHGDH WT or Flag-PHGDH S371A. Cells then were treated with 0.5 mM H_2_O_2_ for 12 h. The serine and glycine contents (**a**), GSH and ROS level (**b**, **c**) in these cells were measured. **d**‒**f** SK-Hep1 cells stably expressing shPHGDH were infected with lentivirus expressing shNT or shMAPK13 and then infected with lentivirus expressing Flag-rPHGDH WT or Flag-rPHGDH S371D respectively. Cells then were treated with 0.5 mM H_2_O_2_ for 12 h. The serine and glycine contents in the cells (**d**), GSH and ROS level (**e,** **f**) in these cells were measured. Data represent means ± SD of three independent experiments. *P* values were determined by two-tailed Student’s *t*-test.

SSP produces glycine for the biosynthesis of reducing GSH. GSH is one of the important endogenous antioxidant, which can maintain cellular redox balance and prevent oxidative damage and cell death^33,34^. To determine whether PHGDH pS371 influences SSP activity and GSH production, we examined the abundance of serine, glycine and GSH in PHGDH-depleted SK-Hep1 cells rescued with rPHGDH WT, S371A or S371D. The cells expressing rPHGDH S371A had much higher levels of serine, glycine and GSH than the cells expressing rPHGDH WT after H_2_O_2_ treatment (Fig. 6a, b). In contrast, the cells expressing rPHGDH S371D had much lower serine, glycine and GSH than the cells expressing rPHGDH WT (Supplementary Fig. S6a, b). Consequently, much lower reactive oxygen species (ROS) levels were detected in the cells expressing rPHGDH S371A than in the cells expressing rPHGDH WT after H_2_O_2_ treatment (Fig. 6c), while much higher ROS levels were observed in the cells expressing rPHGDH S371D than in the cells expressing rPHGDH WT (Supplementary Fig. S6c).

In addition, we depleted PSAT1, the other enzyme in SSP, in SK-Hep1 cells with shRNA (Supplementary Fig. S6d). Compared to control cells, the levels of serine, glycine and GSH were much lower in PSAT1-depleted cells (Supplementary Fig. S6e, f), while ROS levels were much higher in PSAT1-depleted cells after H_2_O_2_ treatment (Supplementary Fig. S6g).

Collectively, these results further demonstrate the important role of serine and glycine synthesis mediated by the serine synthesis pathway in the production of GSH and the alleviation of cellular oxidative stress.

To confirm that MAPK13 inhibits SSP activity, GSH production and redox homeostasis by phosphorylating PHGDH S371, we depleted MAPK13 in PHGDH-depleted SK-Hep1 cells rescued with rPHGDH WT or S371D. MAPK13 depletion increased the levels of serine, glycine and GSH but decreased ROS levels in the cells expressing rPHGDH WT after H_2_O_2_ treatment, but failed to do so in the cells expressing rPHGDH S371D (Fig. 6d‒f). Collectively, these results demonstrate that upon oxidative stress, MPAK13 phosphorylates PHGDH S371 to inhibit SSP and GSH production, which leads to the accumulation of intracellular ROS.

CYP2E1 is responsible for the transformation of NAPQI, and its expression is correlated with the extent of APAP-induced liver injury. We examined the protein levels of CYP2E1 in *Phgdh*^*fl/fl*^ and *Phgdh*^*LKO*^ mice livers with or without APAP treatment. We observed the elevated zonal expression of CYP2E1 after APAP treatment, while there was no obvious difference in CYP2E1 expression between *Phgdh*^*fl/fl*^ and *Phgdh*^*LKO*^ mice (Supplementary Fig. S6h), indicating that PHGDH does not regulate CYP2E1 expression during APAP-induced liver injury.

### Dietary serine supplementation alleviates acute or chronic liver injury

**Fig. 7:**
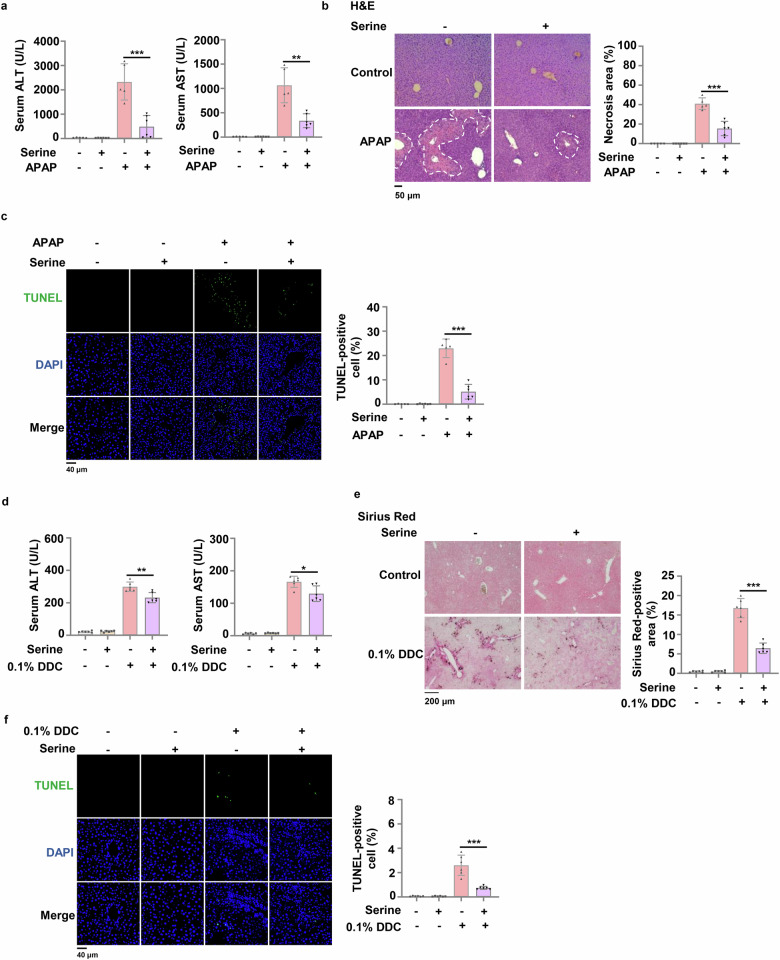
Serine supplementation alleviates oxidative stress-associated acute or chronic liver injury. **a**–**c** c57BL/6J mice were intraperitoneally injected with normal saline or 300 mg/kg APAP for 24 h. And at 0 h and 12 h, mice were treated with 200 mg/kg serine solution or normal saline (control) by intragastric administration (i.g.). Serum ALT and AST levels were detected by kit (**a**). Liver necrosis area in H&E-stained sections were circle and quantified (**b**). Positive liver cells in TUNEL-stained sections were quantified (**c**). Representative images of H&E staining or TUNEL staining were shown on the left panel and statistical analysis were shown on the right panel (*n* = 5 in control group and *n* = 6 in serine supplementation group). **d**‒**f** c57BL/6 J mice were fed with 0.1% DDC irradiated diet with or without 10% serine for 2 weeks. Serum ALT and AST levels were detected (**d**). Sirius Red staining (**e**) and TUNEL staining (**f**) were performed in liver sections from these mice (*n* = 6 per group). Data are means ± SD. Each point represents an individual mouse. *P* values were determined by two-tailed Student’s *t*-test.

It has been demonstrated earlier that SSP is suppressed during liver injury due to the degradation of PHGDH, causing a drop in its downstream metabolites like serine, glycine and GSH, which results in hepatocyte death due to oxidative stress. Thus, we wondered whether dietary serine supplementation alleviates liver damage. Serine supplementation increased the levels of serine in serum and the levels of serine and glycine in livers after APAP or DDC treatment (Supplementary Fig. S7a‒d). More importantly, the levels of ALT and AST in serum were decreased, the necrosis in hepatic lobule was alleviated and less dead cells were observed in the liver tissues dissected from APAP-treated mice administrated with dietary serine (Fig. 7a‒c).

In addition, we observed that serine supplementation also markedly decreased the levels of ALT and AST in serum of the mice treated with DDC (Fig. 7d). Furthermore, liver fibrosis was also relieved, as indicated by Sirius Red staining, and less dead cells were observed in DDC-treated mice after serine supplementation (Fig. 7e, f).

Collectively, these results indicate that dietary serine supplementation can effectively alleviate APAP- or DDC-induced liver injury, suggesting the therapeutic potential of serine supplementation for the treatment of oxidative stress-associated acute or chronic liver injury.

## Discussion

According to previous data, DILI accounts for a significant proportion of acute liver failure cases in the United States and is a leading cause of drug withdrawal from the market^2^. Many drugs, such as antineoplastic agents, acetaminophen (paracetamol), antibiotics and herbal and dietary supplements, are commonly associated with DILI^5^. Understanding the mechanisms underlying the process of DILI will ultimately contribute to the development of biomarkers for early detection and the identification of new target molecules to intervene DILI process. In this study, we reveal the protective role of SSP in drug-induced acute or chronic liver injury and demonstrate the regulatory mechanism of SSP during damage process, in which MAPK13 phosphorylates PHGDH, the rate-limiting enzyme of SSP, at S371 upon oxidative stress and activates CMA-mediated protein degradation of PHGDH, thereby inhibiting SSP flux. Decreased SSP flux leads to reduced GSH synthesis and oxidative stress-induced cell death of hepatocytes, thereby exacerbating DILI. Meanwhile, we also confirm that MAPK13 inhibition and dietary serine supplementation can mitigate oxidative stress-associated acute or chronic liver injuries, highlighting the therapeutic potential of MAPK13 inhibitor and dietary serine supplementation (Supplementary Fig. S7e).

It has been shown that the process of liver injury is accompanied with extensive metabolic reprogramming and reprogrammed metabolic pathway contributes to the progression of liver injuries caused by various stresses. A recent study indicates that reduced glycine levels caused by reversed SHMT2 activity in mice with hepatic steatosis exacerbate acetaminophen-induced liver injury^9^. Another study indicates that upregulated kynurenine aminotransferases 2 following hepatic ischemia-reperfusion (IR) shifts the kynurenine metabolic route from 3-HAA and QA to KYNA synthesis impairing de novo nicotinamide adenine dinucleotide biosynthesis, which promotes liver injury^35^. In contrast, how metabolism is reprogrammed during DILI remains unclear. Here we show that the flux of SSP is inhibited during DILI, caused by the degradation of its rate-limiting enzyme PHGDH. Inhibited SSP leads to reduced GSH levels and increased ROS levels, thereby exacerbating DILI. Importantly, we further demonstrate that PHGDH degradation is triggered by MAPK13-mediated PHGDH S371 phosphorylation, and that MAPK13 inhibition can recover PHGDH protein levels, the flux of PHGDH-regulated SSP and GSH production, thereby protecting hepatocytes from oxidative stress-induced cell death and dampening the progression of DILI. Based on the mechanism underlying rewired SSP in DILI, two therapeutic strategies have been proposed to treat DILI, including MAPK13 inhibition and dietary serine supplementation.

The p38MAPK family plays a central role in orchestrating the cellular response to stress and inflammation^36^. As an important member of the family, MAPK13 has been shown to promote DOX-induced cardiotoxicity by inhibiting autophagy^37^. Similarly, MAPK13 could also exacerbate acute lung injury by conversely regulating PTEN activity in neutrophils with PKD1^38^. However, the role of MAPK13 in liver injury and how MAPK13 regulates the process of the injury remain unknown. In our study, we found that the deficiency of MAPK13 ameliorates oxidative stress-induced liver injury, which is expected to provide a new target for liver injury treatment. Meanwhile, we uncovered the mechanism of MAPK13-promoted liver injury. During the process of liver injury, MAPK13 phosphorylates PHGDH upon oxidative stress and promotes its autophagic degradation, thereby inhibiting SSP flux and GSH production, which leads to the necrosis of hepatocytes. It also revealed the new mechanism of the regulation of PHGDH by MAPK13.

PHGDH can be regulated via multiple sets of mechanisms. It has been shown that the expression of PHGDH is regulated by many transcription factors, such as HOXA10, SMAD3 and ATF3, in endometrial endothelial cells, pulmonary epithelial cells and colon cancer cells^39–41^. Besides, the enzymatic activity of PHGDH could also be enhanced by CUL4A‒DDB1 complex-mediated monoubiquitination to promote colorectal cancer (CRC) metastasis, inhibited by PKCζ-mediated phosphorylation to dampen CRC progression, or enhanced by PRMT1-mediated methylation to promote hepatocellular carcinoma progression^42–44^. In contrast, how PHGDH protein stability is regulated remains unexplored. In this study, we demonstrate that during acute or chronic liver injury, PHGDH is phosphorylated by MAPK13 upon oxidative stress, which initiates CMA-mediated protein degradation of PHGDH, thereby sensitizing hepatocytes to oxidative stress and exacerbating liver injury.

## Materials and methods

### Antibodies and reagents

Primary antibodies were used against: PHGDH (14719-1-AP, Proteintech Group); Actin (60008-1-lg, Proteintech Group); p53 (sc-126, Santa Cruz Biotechnology); Tubulin (T9026, Sigma-Aldrich); LAMP-2 (sc-18822, Santa Cruz Biotechnology); HSC70 (sc-7298, Santa Cruz Biotechnology); Flag (20543-1-AP, Proteintech Group); HA (3724S, Cell Signaling Technology); p38δ MAPK (MAPK13) (2308, Cell Signaling Technology); p38 MAPK T180/Y182 phosphorylation (4511, Cell Signaling Technology); PHGDH (A10461, Abclonal); CYP2E1 (19937-1-AP, Proteintech Group); PHGDH S371 phosphorylation (customized, Abclonal). The following secondary antibodies were used: goat-anti-mouse IgG second antibody (31160, Thermo) and goat-anti-rabbit IgG second antibody (31210, Thermo). The primary antibodies were used at a 1:1000 dilution for immunoblotting and a 1:200 dilution for IHC. Secondary antibodies were used at 1:3,000 dilution for immunoblotting. Puromycin (540222) and hygromycin (400053) were bought from EMD Biosciences. [γ-32P] ATP (NEG502A) was purchased from PerkinElmer. MAPK13-IN-1 (HY-18850) was purchased from MCE. Gossypetin (HY-119917) was bought from MCE. L-Serine (ST1512; S0010) was purchased from Beyotime and Solarbio. 3,5-Diethoxycarbonyl-1,4-dihydro-2,4,6-collidine (DDC) (137030) was purchased from Sigma-Aldrich. Acetaminophen (HY-66005) and Cycloheximide (HY-12320) were bought from MCE. Sirius rosa BB (S8060) was bought from Solarbio. Chloroquine (S6999) and MG132 (S2619) were purchased from Selleckchem. DeadEnd™ Fluorometric TUNEL System (G3250) was bought from Promega. TUNEL assay kit (C1086) was bought from Beyotime. Reduced GSH Content Assay Kit (BC1175) was bought from Solarbio. AST (C010-2-1) and ALT (C009-2-1) assay kits were purchased from NJJCBio. DNA transfection reagent was purchased from Signagen Laboratories. Lipofectamine 3000 (L3000015) was bought from Thermo Fisher Scientific.

### DNA constructs and mutagenesis

PCR-amplified human *PHGDH* was cloned into pCDH-Flag, pCDH-SFB, pCMV-Flag or pCold I-His. PCR-amplified mouse *Phgdh* was cloned into pCMV-Flag. PCR-amplified human *MAPK13* was cloned into pcDNA3-HA or pColdI-His. PCR-amplified mouse *Mapk13* was cloned into pcDNA3-HA. PCR-amplified human *HSC70* was cloned into pcDNA3-HA. Human *PHGDH*, mouse *Phgdh* and human *MAPK13* mutations were made using the QuikChangeIISite-Directed Mutagenesis Kit (Stratagene).

The pGIPZ shNT was generated with the control oligonucleotide 5′-GCTTCTAACACCGGAGGTCTT-3′. pGIPZ human *PHGDH* shRNA was generated with 5′-CACGACAGGCTTGCTGAATGA-3′ oligonucleotide. pGIPZ human *MAPK13* shRNA was generated with 5′-CAGCCGTTTGATGATTCCTTA-3′ oligonucleotide. pGIPZ human *HSC70* shRNA was generated with 5′-CCTGATGAAGCTGTTGCTTAT-3′ oligonucleotide. pGIPZ human *LAMP2A* shRNA was generated with 5′-AAGCTGGAACCTATTCAGTTA-3′ oligonucleotide. pGIPZ human *PSAT1* shRNA (#1) was generated with 5′-ACATACTTTATATATGTTTAT-3′ oligonucleotide. pGIPZ human *PSAT1* shRNA (#2) was generated with 5′-GCCAAGAAGTTTGGGACTATA-3′ oligonucleotide. siRNA of mouse *Mapk13* was 5′-GGUCUAAAGUACAUCCACUTT-3′ oligonucleotide (bought from GenePharma). Primers were synthesized from Genewiz. Some other primers for real-time PCR were also synthesized from Genewiz and the primer sequences were presented in Supplementary Table S1.

### Cell culture

SK-Hep1 and HEK293T cell lines were maintained in high glucose Dulbecco’s modified Eagles’s medium (DMEM) supplemented with 10% fetal bovine serum (FBS). Cell lines were obtained from the Cell library of the Chinese Academy of Sciences. Primary hepatocytes isolated from mice were maintained in the DMEM/F-12 medium with 10% FBS. The cell lines were authenticated using the short tandem repeat (STR) method and all the cells were incubated in 5% CO_2_ at 37 °C.

### Animal studies

All mice were housed in a specific pathogen-free environment and treated in strict accordance with protocols approved by the Institutional Animal Use Committee of the Center for Excellence in Molecular Cell Science, Chinese Academy of Science (number for approval: SIBCB-S355-2312-39). WT (C57BL/6J background) mice were obtained from Shanghai Experimental Animal Center. *Mapk13* knockout mice (C57BL/6J background) and *Phgdh*^*fl/fl*^ mice (C57BL/6 J background) were purchased from Shanghai Model Organisms Center. Alb-cre mice were gently provided by Lei Zhang.

### Cell viability assay

SK-Hep1 with or without genetic modifications were plated in 12-well plates and were treated with H_2_O_2_. Cell viability was determined by trypan blue staining. Primary hepatocytes isolated from mice were seeded in 12-well plates and treated with H_2_O_2_ for 24 h. Cell death was quantified after PI and Hoechst staining. Fluorescence images were obtained using an Olympus BX53 microscope.

### Purification of recombinant proteins

His-PHGDH, His-PHGDH S371A mutant and MAPK13 active mutant (His-MAPK13 F324S) were expressed in bacteria and purified. Briefly, all the three plasmids were transformed to BL21/DE3 bacteria separately. Protein expression was induced by using 0.5 mM IPTG for 18 h at 16 °C. The cell pellets were collected and were crushed by high-pressure breaking in PBS (pH 7.4). Then the lysis was collected by centrifugation at 12,000 rpm for 60 min (4 °C). Cleared lysates were then bound to Ni-NTA resin (Gene script) for 2 h, with rolling at 4 °C. Beads were washed extensively before eluting for 1 h in PBS with 500 mM imidazole. Eluted proteins were then concentrated against 25 mM Tris-HCl pH 7.4, 10 mM MgCl_2_. The protein was stored in 50% glycerin in ‒80 °C.

### In vitro kinase assay

The bacterially purified His-PHGDH or His-PHGDH S371A (1 μg) were incubated with His-MAPK13 F324S (0.5 μg) in the kinase buffer (25 mM Tris-HCl pH 7.4, 5 mM β-Glycerophosphate, 0.1 mM Na_3_VO_4_, 10 mM MgCl_2_, 2 mM DTT, 5 μCi ^32^P-labeled ATP) in a total volume of 30 μL at 30 °C for 30 min. The reactions were terminated by the addition of SDS-PAGE loading buffer.

### Isolation of primary hepatocytes

A two-step collagenase perfusion procedure was used to isolate primary mouse hepatocytes. Briefly, 8‒10-week-mice were anesthetized with avertin and then were perfused through portal vein cannulation by 50 mL HBSS containing 20 mM HEPES and 5 mM EGTA (Step1), followed by treatment with the enzymatic solution HBSS (50 mL) containing 5 mM CaCl_2_ and 3000 U collagenase I (Step2). The cell suspensions were filtered through a 70 μm mesh and then centrifuged at 50× *g* for 5 min at 4 °C. After splitting of red cells, the live primary hepatocytes were separated with 50% SIP after centrifugation at 400× *g* for 10 min. Collected hepatocytes were washed with PBS twice and then counted and seeded in Geltrex™ Basement Membrane Matrix coating dish.

### Induction of APAP toxicity

To induce APAP toxicity, male mice at 8‒10-week old fasted for 16 h were injected intraperitoneally with 250 mg/kg or 300 mg/kg APAP. Mice were sacrificed at indicated time and livers were collected. Blood was collected from heart. And to test the survival during 72 h, the mice were injected with a lethal dose of 750 or 800 mg/kg and the number of died mice was recorded every 6‒12 h.

### Cholestatic liver injury model

To induce chronic liver injury, 8‒10-week mice were fed a diet containing 0.1% 3,5-diethoxycarbonyl-1,4-dihydrocollidine (DDC) for two weeks. Then the liver tissue and blood samples were collected for analysis.

### Serum AST and ALT measurement

Mouse blood after APAP or DDC diet treatment was collected and stored at 4 °C for 3 h. After that, the samples were centrifuged at 3500 rpm for 15 min at 4 °C. Transferring the serum on the upper layer to a new 1.5 mL centrifuge tube. The serum was collected for measurement of alanine aminotransferase and aspartate aminotransferase activity as the manufacturer’s protocol of ALT and AST assay kit.

### TUNEL staining

For TUNEL staining, the tissue sections from paraffin-embedded mouse livers were stained with the TUNEL kit as the manufacture’s protocol. Tissue imaging was performed on a Leica TCS SP8 confocal laser scanning microscope (software LAS X). Three to five fields per tissue section were randomly selected under microscopy. And the percentage of positive cell was calculated. An average of these fields was used to represent each case.

### Sirius Red staining

Firstly 0.1 g Sirius rosa BB was dissolved in 100 mL of saturated picric acid solution. Paraffin-embedded liver tissue sections from mice after 0.1% DDC treatment were dewaxed to water, and then stained with the solution for 30 min. Then the sections were differentiated by anhydrous ethanol and blocked with neutral balsam. Three to five fields per tissue section were randomly selected under microscopy. The fibrosis area of each field was calculated. An average of these fields was used to represent each case.

### IHC analysis

The tissue sections from paraffin-embedded mice liver after APAP treatment were stained with anti-PHGDH pS371 and anti-CYP2E1 antibodies. Three to five representative fields per tissue section were randomly selected under microscopy. The tissue sections were quantitatively scored according to the percentage of positive cells and staining intensity. The intensity of staining was rated on a scale of 0‒3: 0, negative; 1, weak; 2, moderate; and 3, strong. The following proportion scores were assigned: 0 if 0% of the cells showed positive staining, 1 if 0%‒1% of cells were stained, 2 if 2%‒10% were stained, 3 if 11%‒30% were stained, 4 if 31%‒70% were stained, and 5 if 71%‒100% were stained. The proportion and intensity scores were then combined to obtain a total score (range, 0‒8). An average of the scores of representative fields per tissue section was used to represent each case.

### ROS detection

ROS in cells were detected with Reactive Oxygen Species Assay kit as the manufacture’s protocol. In short, cells were seeded in 6-well plates overnight. Then the cells were collected after 0.5 mM H_2_O_2_ treatment for 12 h. ROS probe (DCFH-DA) was diluted to 10 μM with DMEM. Collected cells were incubated in diluted probe buffer at 37 °C for 20 min. After that cells were washed with DMEM for three times and the ROS concentration of cells were detected with flow cytometer (Cytoflex LX) in FITC channel. Fluorescence intensity represents the ROS content in cell.

### GSH measurement

GSH level of cell or liver tissue was measured with the Glutathione content detection kit as the manufacture’s protocol. Briefly, for liver tissue after APAP treatment, 0.1 g liver tissue was added to 1 mL buffer 1 and homogenized on ice for several seconds. For cultured cells after H_2_O_2_ treatment, cells were collected and washed with PBS. And cell pellet was resuspended with 1 mL buffer 1, which then were frozen (in liquid nitrogen) and melted (in 37 °C water bath) for 2‒3 times. After that, tissue or cell samples were centrifuged at 8000× *g* for 10 min at 4 °C and the supernatant was collected to measure. The 412 nm absorbance represents the content of samples after reaction with the buffer 2 and buffer 3 comparing with GSH standard curve.

### Liquid Chromatography-Mass Spectrometry (LC-MS)-based untargeted metabolomics

8 weeks c57BL/6 J mice were intraperitoneally injected with 250 mg/kg APAP. Liver tissues were collected at 0 h, 12 h, 24 h and 36 h. 200 µL of H_2_O was added to 20 mg tissue, and the tissue was homogenized under low temperature. 800 µL of MeOH:ACN (v:v, 1:1) was added to 200 µL of tissue homogenate. The mixture was sonicated for 10 min at 4 °C in water bath and then incubated for 1 h at ‒20 °C. The supernatant was collected by centrifuging for 15 min at 13,000 rpm and 4 °C. Using a vacuum concentrator to evaporate the supernatant to dryness. Before LC-MS analysis, reconstituting dried samples with 100 µL of ACN: H_2_O (v:v, 1:1). After sonicating for 10 min at 4 °C in water bath and centrifuging for 15 min at 13,000 rpm and 4 °C, the supernatant was transferred into LC sample vials for LC-MS analysis.

We used a UHPLC system (Vanquish, Thermo Scientific) coupled to an orbitrap mass spectrometer (Exploris 480, Thermo Scientific) to acquisite data. Waters ACQUITY UPLC BEH Amide column (particle size, 1.7 µm; 100 mm (length) × 2.1 mm (i.d.)) and Phenomenex Kinetex C18 column (particle size, 2.6 µm; 100 mm (length) × 2.1 mm (i.d.)) were used for LC separation for HILIC mode and RP mode, respectively. The temperature was kept at 25 °C. For HILIC mode, mobile phases A was 25 mM ammonium acetate and 25 mM ammonium hydroxide in 100% water, while B was 100% acetonitrile used for both ESI positive and negative modes. The linear gradient eluted from 95% B (0.0‒0.5 min), 95% B to 65% B (0.5‒7.0 min), 65% B to 40% B (7.0‒8.0 min), 40% B (8.0‒9.0 min), 40% B to 95% B (9.0‒9.1 min), then stayed at 95% B for 2.9 min. The flow rate was 0.5 mL/min. The sample injection volume was 2 µL. For RP mode, mobile phases A was 0.01% acetic acid in 100% water, while B was acetonitrile/isopropanol (1/1; v/v) used for both ESI positive and negative ionization modes. The linear gradient eluted from 1% B (0.0‒1.0 min), 1% B to 99% B (1.0‒8.0 min), 99% B (8.0‒9.0 min), 99% B to 1% B (9.0‒9.1 min), then stayed at 95% B for 2.9 min. The flow rate was 0.3 mL/min. The sample injection volume was 2 µL. ESI source parameters were set as follows: spray voltage, 3500 V or ‒2800 V, in positive or negative modes, respectively; aux gas heater temperature, 350 °C; sheath gas, 50 arb; aux gas, 15 arb; capillary temperature, 400 °C. LC-MS data acquisition was operated under full scan polarity switching mode for all samples. A ddMS2 scan was applied for QC samples to acquire MS/MS spectra. The full scan was set as orbitrap resolution, 60,000; AGC target, 1e6; maximum injection time, 100 ms; scan range, 70‒1200 Da. The ddMS2 scan was set as orbitrap resolution, 30000; AGC target, 1e5; maximum injection time, 60 ms; scan range, 50-1200 Da; top N setting, 6; isolation width, 1.0 *m/z*; collision energy mode, stepped; collision energy type, normalized; HCD collision energies (%), SNCE 20-30-40%; Dynamic exclusion duration was set as 4 s for excluding after 1 time.

The metabolomics data processing followed Zheng-Jiang Zhu’s previous publications^45^. In brief, the raw MS data (.raw) files were converted into the mzXML format by ProteoWizard (version 3.0.20360) and processed by XCMS (version 3.2; https://bioconductor.org/packages/release/bioc/html/xcms.html) for peak detection, retention time correction, and peak alignment. Metabolite annotation was performed using MetDNA (http://metdna.zhulab.cn/)^46^. The metabolite annotation parameters were set as “HILIC” or “RP” according to liquid chromatography mode, and “30” or “SNCE_20_30_40%” for collision energy. Comparing the metabolites at different time points with 0 h after APAP treatment, we select and map the metabolites that changed commonly at the three time points to KEGG Pathway database (http://www.kegg.jp/kegg/pathway.html). Significantly enriched pathways are identified with Fisher’s Exact Test for a given list of metabolites.

### Mass spectrometry analysis of glycine and serine

Cultured cells (10 cm dish) were washed with PBS and immediately incubated with 1 mL ice-cold methanol: acetonitrile: water (40:40:20) with 0.5% formic acid on ice for 5 min. 50 µL 15% NH_4_HCO_3_ was then added. The cells were scraped and centrifuged at 15,000× *g* for 10 min at 4 °C. The supernatant was then transferred to clean tubes and evaporated to dryness under nitrogen. For liver tissue, 50 mg tissue was added to 500 µL of 80% (v/v) HPLC-grade methanol (pre-chilled at ‒80 °C). Then we ground them for 1‒2 min with tissue grinder on dry ice in the tube, vortexed them for 1 min at 4‒8 °C and incubated them at ‒80 °C overnight. The supernatant was then transferred to clean tubes after centrifuge at 14,000× *g* for 20 min using a refrigerated centrifuge at 4 °C. The supernatant was transferred to clean tubes and evaporated to dryness under nitrogen. Before LC-MS analysis, cell metabolite pellet was re-dissolved in 200 µL methanol:acetonitrile:water (40:40:20) and tissue metabolite pellet was re-dissolved in 200 µL of 80% (v/v) HPLC-grade methanol at 14,000× *g* for 10 min at 4 °C. Finally, the supernatant was stored at ‒80 °C briefly or performed LC-MS analysis directly.

LC-MS/MS analysis was conducted using LC-MS system comprising the Agilent 1290 Infinity II UHPLC system tandem with Agilent 6495 mass spectrometer. Chromatographic separation was achieved on ACQUITY UPLC BEH Amide column (100 mm × 2.1 mm, 1.7 μm) at 40 °C. The mobile phase consisted of 5 mM ammonium acetate, 0.1% FA in water (A) and 0.1% FA in acetonitrile (B) at a flow rate of 0.3 mL/min. The column was eluted with 90% mobile phase B for 1 min, followed by a linear gradient to 50% mobile-phase B over 9 min, held at 50% for 4 min, a linear gradient to 90% mobile phase B over 0.5 min, then 3.5 min at 90% mobile-phase B. The sample volume injected was 5 µL. Mass spectrometer operating in positive ion mode using the following settings: sheath gas temperature 250 °C, sheath gas flow 11 L/min, Capillary 3000 V, Nozzle voltage 1500 V, gas temperature 200 °C, Gas flow 14 L/min, Nebulizer 20 psi. Compounds were measured by multiple reaction monitoring with optimized collision energy are shown in the table.

**Table:** 

Compound	Precursor ion (Da)	Product ion (Da)	collision energy (V)
Serine	106.10	88	9
Serine	106.10	60	9
Glycine	76.04	30.1	13

### Mass spectrometry analysis of phosphorylation

SFB-PHGDH was pulled down from SK-Hep1 cell treated with H_2_O_2_ treatment for 0 h or 3 h. The precipitated complexes were boiled at 95 °C for 10 min. SFB-PHGDH (for detection of PHGDH phosphorylation) proteins were separated from the complexes using SDS-PAGE gel. As for the analysis of PHGDH phosphorylation after in vitro kinase assay, the reaction system with or without His-MAPK13 F324S incubation was also boiled at 95 °C for 10 min after the reaction. His-PHGDH proteins were separated from the system using SDS-PAGE gel. The Coomassie Brilliant Blue stained gel bands were cut and stored at ‒80 °C.

#### In-gel digestion and mass spectrometry analysis

The Coomassie Brilliant Blue stained gel band was excised into small pieces and washed in order with water, 50 mM NH_4_HCO_3_ in 50% acetonitrile and 100% acetonitrile. The protein was reduced with 10 mM TCEP (Thermo Scientific) in 100 mM NH_4_HCO_3_ at room temperature for 30 min and alkylated with 55 mM iodoacetamide (Sigma) in 100 mM NH_4_HCO_3_ in the dark for 30 min. After that, the gel pieces were washed with 100 mM NH_4_HCO_3_ and 100% acetonitrile, and then dried using a SpeedVac. Finally, they were digested with 12.5 ng/μL trypsin (Promega) in 50 mM NH_4_HCO_3_ for 16 h at 37 °C, and the tryptic peptides were extracted twice with 50% acetonitrile/5% formic acid and dried using a SpeedVac. The sample was reconstituted with 0.1% formic acid, desalted using a MonoSpinTM C18 column (GL Science, Tokyo, Japan), and then dried with a SpeedVac.

#### LC/tandem MS (MS/MS) analysis of peptides

The peptide mixture was loaded onto an Easy-nLC1200 system equipped with a home-made reverse phase C18 column (75 µm × 30 cm, 1.9 µm) with a 120 min gradient from 2% to 100% of buffer B (buffer A: 0.1% formic acid in water; buffer B: 0.1% formic acid in 80% Acetonitrile) at 300 nL/min. The eluted peptides were ionized and directly introduced into a Q-Exactive mass spectrometer (Thermo Scientific, San Jose, CA) using a nano-spray source with the application of a distal 2.5-kV spray voltage. A cycle of one full-scan MS spectrum (*m/z* 300‒1800) was acquired followed by top 20 MS/MS events, sequentially generated on the first to the twentieth most intense ions selected from the full MS spectrum at a 28% normalized collision energy. MS scan functions and LC solvent gradients were controlled by the Xcalibur data system (Thermo Scientific).

#### Data analysis

The acquired MS/MS data were analyzed against a human database (including all target proteins) using PEAKS Studio 8.5. Cysteine alkylation by iodoacetamide was specified as fixed modification with mass shift 57.02146 and Methionine oxidation was set as variable. Additionally, serine/threonine phosphorylation was set as dynamic modification with mass shift 79.9663. A decoy database containing the reversed sequences of all the proteins was appended to the target database to accurately estimate peptide probabilities and false discovery rate (FDR), and FDR was set at 0.01.

### Statistical analysis

All the data were obtained from three or more independent repeated experiments. The number of animals is specified in each figure legend. Data are presented as means ± SD. Survival-related statistics were analyzed using the log-rank test, and the remaining analyses were performed using the unpaired two-tailed Student’s *t*-test. Statistical significance is indicated as follows: ns, not significant; *P < 0.05; **P < 0.01; ***P < 0.001; ****P < 0.0001.

## Supplementary information

Supplementary figures
