# Supplementary material for: MAPK13 phosphorylates PHGDH and promotes its degradation via chaperone-mediated autophagy during liver injury
Source: Cell Discov. 2025 Feb 18;11:15. doi: 10.1038/s41421-024-00758-w (PMC11832932; doi:10.1038/s41421-024-00758-w)
Supplement: Supplementary file 1 — Supplementary figures [file 41421_2024_758_MOESM1_ESM.pdf]

MAPK13 phosphorylates PHGDH and promotes its degradation via chaperone-mediated autophagy during liver injury

Ru Xing,[1,5] Ruilong Liu,[2,5] Yongxiao Man,[1,5] Chen Liu,[1] Yajuan Zhang,[4] Hong Gao[1] and Weiwei Yang[1,3]*

[1]Key Laboratory of Multi-Cell Systems, Shanghai Key Laboratory of Molecular Andrology, Shanghai Institute of Biochemistry and Cell Biology, Center for Excellence in Molecular Cell Science, University of Chinese Academy of Sciences, Chinese Academy of Sciences, Shanghai, China

[2]Ben May Department for Cancer Research, The University of Chicago, Chicago, IL, USA

[3]School of Life Science, Hangzhou Institute for Advanced Study, University of Chinese Academy of Sciences, Hangzhou, Zhejiang, China

[4]Shanghai Institute of Thoracic Oncology, Shanghai Chest Hospital, Shanghai Jiao Tong University School of Medicine, Shanghai, China

[5]These authors contributed equally

*Correspondence: wyang@sibcb.ac.cn (W.Y.)

Supplementary Figures

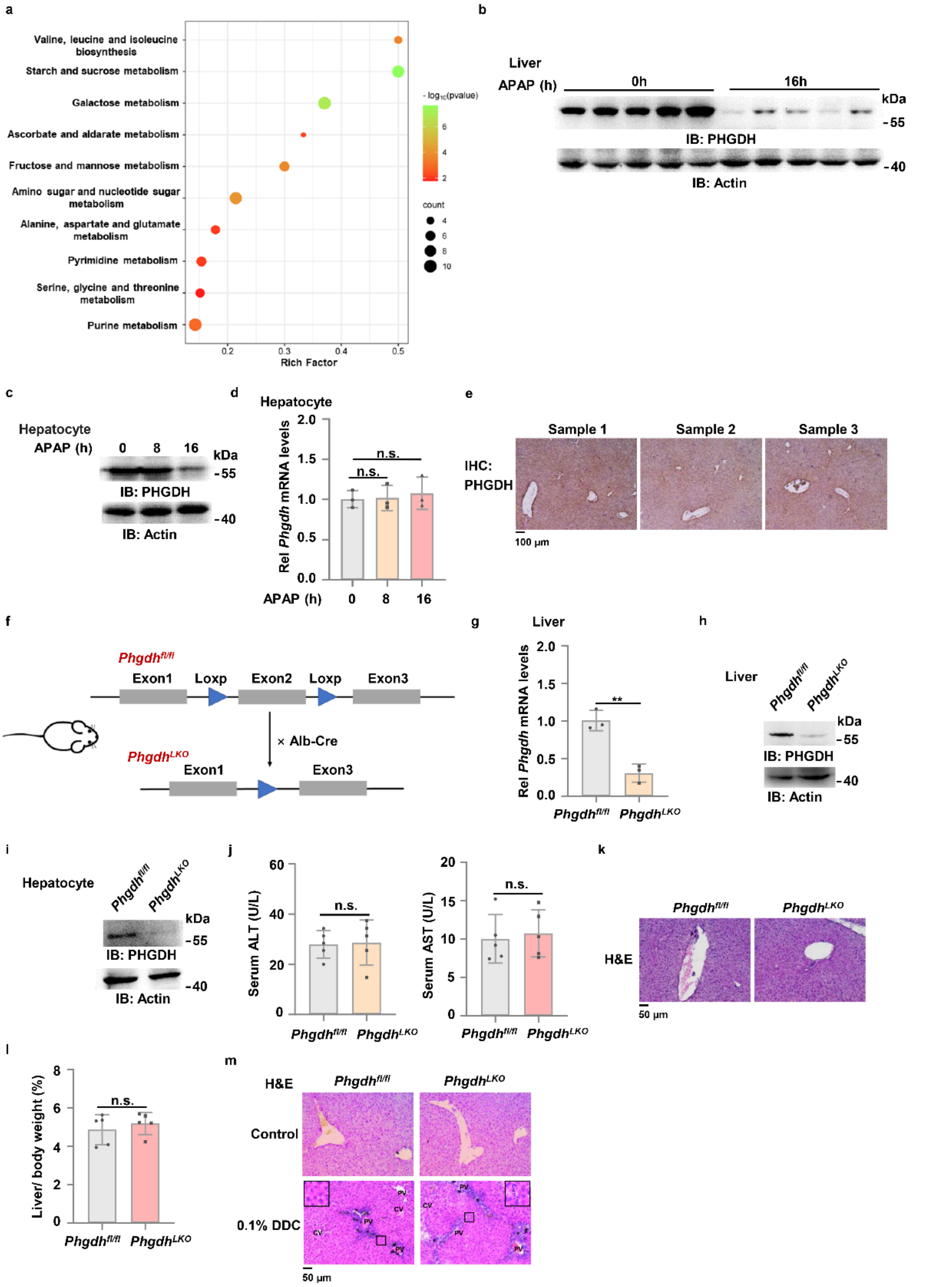

Supplementary Fig. S1 Acetaminophen decrease the protein level of PHGDH in liver, related to Fig. 1.

a. c57BL/6J mice were intraperitoneally injected with 250 mg/kg APAP. Livers were collected at 0 h, 12 h, 24 h, 36 h after APAP treatment, and the tissue samples were used for untargeted metabolomics mass spectrometry. We analyzed the differential metabolites at different time

points compared with 0 h, and selected the common differential metabolites for KEGG pathway enrichment analysis (n = 6 in each time point).

b. c57BL/6J mice were intraperitoneally injected with 250 mg/kg APAP. Livers were collected at 0 h or 16 h after APAP treatment (n = 5 in each time point). IB, immunoblotting.

c. Primary hepatocytes from c57BL/6J mice were treated with 20 mM APAP for 0 h, 8 h or 16 h. Immunoblotting analysis was performed.

d. Primary hepatocytes from c57BL/6J mice were treated with 20 mM APAP for 0 h, 8 h or 16 h. *Phgdh* mRNA levels were examined in these cells (n = 3).

e. IHC staining of dissected livers from three c57BL/6J mice with anti-PHGDH antibody were performed.

f. Schematic model of strategy of *Phgdh* specifically knocked out in hepatocytes of mice.

g, h. mRNA levels (**g**, n = 3) and protein levels (**h**) of PHGDH in livers of *Phgdh*$^{fl/fl}$ and *Phgdh*^{LKO} mice were examined.

i. PHGDH protein levels in primary hepatocytes derived from *Phgdh*$^{fl/fl}$ and *Phgdh*^{LKO} mice were examined.

j. Serum ALT and AST levels of *Phgdh*$^{fl/fl}$ and *Phgdh*^{LKO} mice were shown (n = 5 per group).

k. Representative H&E staining of livers from *Phgdh*$^{fl/fl}$ and *Phgdh*^{LKO} mice were shown (n = 5 per group).

l. Liver/ body weight ratio of *Phgdh*$^{fl/fl}$ and *Phgdh*^{LKO} mice were shown (n = 5 per group).

m. Representative H&E staining of livers dissected from *Phgdh*$^{fl/fl}$ and *Phgdh*^{LKO} mice fed with irradiated diet with or without 0.1 % DDC for 2 weeks (n = 6 per group).

Immunoblots are representative of three independent experiments. Data are means ± SD. Each point or lane represents an individual mouse. *P* values are determined by two-tailed Student's *t*-test.

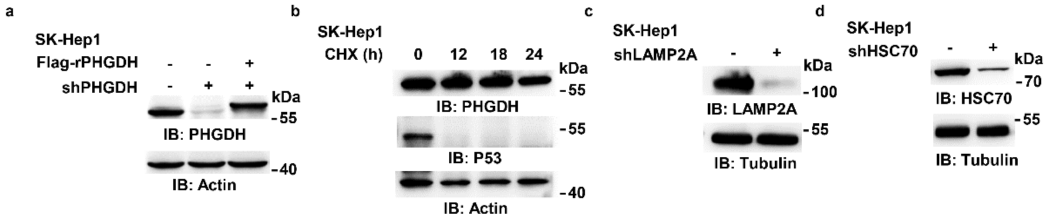

Supplementary Fig. S2 PHGDH is degraded via CMA pathway in hepatocytes upon oxidative stress, related to Fig. 2.

a. SK-Hep1 cells stably expressing shNT or shPHGDH were reconstituted with or without Flag-rPHGDH, and protein expression was detected with indicated antibodies. IB, immunoblotting.

b. SK-Hep1 cells were treated with 50 μg/mL CHX as indicated time.

c, d. SK-Hep1 cells were infected with lentivirus expressing shNT, shLAMP2A or shHSC70.

Immunoblots are representative of three independent experiments.

a

Phospho-Peptides	$-H_2O_2$	$+H_2O_2$
	Phosphorylated intensity/ Unphosphorylated intensity	Phosphorylated intensity/ Unphosphorylated intensity
TSLKNAGNCLs^{371}PAVIVGLLKE	0	2.01×10^{-4}
LGTLMRAWAGs^{349}PKGTIQVITQ	0	1.06×10^{-4}

b

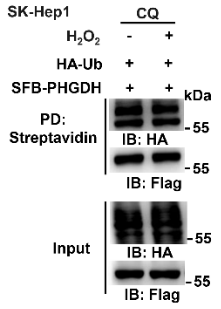

c

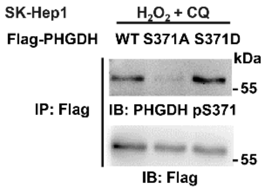

d

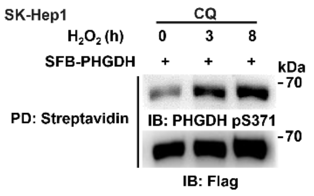

e

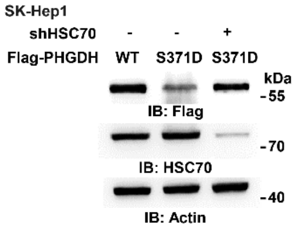

f

Kinase	Kinase group	Log_2(score)	Site percentile	Percentile rank
NLK	CMGC	3.606	97.432%	1
ERK5	CMGC	3.272	97.208%	2
SMG1	PIKK	1.348	96.490%	3
P38D	CMGC	5.682	96.015%	4
P38A	CMGC	4.847	95.888%	5

g

Interactor	Experiments	Interaction score	Confidence
GAK	1	0.536	High
MK06	1	0.508	High
CDK2	1	0.479	High
M3K3	1	0.479	High
STK4	1	0.445	High
MAPK13 (P38D)	1	0.445	High

h

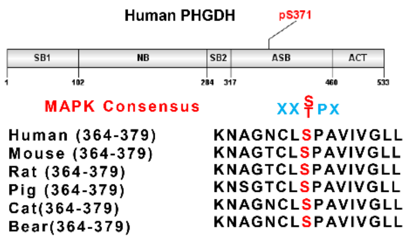

i

Phospho-Peptides	With His-MAPK13	Without His-MAPK13
	Phosphorylated intensity/ Unphosphorylated intensity	Phosphorylated intensity/ Unphosphorylated intensity
PWIGLAEALGt^{341}LMRAWAGSPK	0	6.56×10^{-4}
TSLKNAGNCLs^{371}PAVIVGLLKE	0	2.60×10^{-4}
ANLRKVLISDs^{14}LDPCCRKILQ	0	2.85×10^{-5}
DEGALLRALQs^{251}GQCAGAALDV	0	2.79×10^{-5}
KFMGTELNGKt^{147}LGILGLGRIG	0	8.90×10^{-6}
MRAWAGSPKGt^{353}IQVITQGTSL	0	5.21×10^{-6}

j

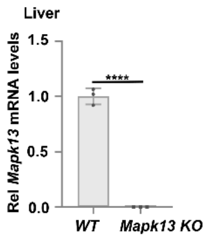

k

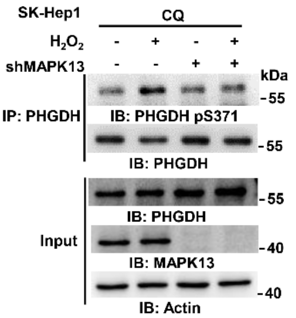

l

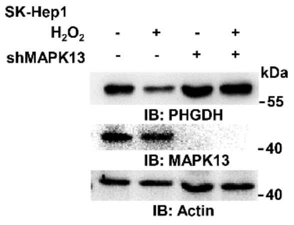

Supplementary Fig. S3 Oxidative stress induces PHGDH pS371 and promotes its degradation, related to Fig. 3.

a. SFB-PHGDH was pulled down from SK-Hep1 cells stably expressing SFB-PHGDH treated with or without 0.5 mM H_2O_2 and 20 μM CQ for 3 h. The phosphorylated residues detected after H_2O_2 treatment were presented. And the results of phosphorylated peptides intensity/ unphosphorylated peptides intensity was shown.

b. SK-Hep1 cells stably expressing SFB-PHGDH were transiently transfected with HA-Ub. SFB-PHGDH was pulled down from the cells treated with or without 0.5 mM H_2O_2 and 20 μM CQ for 8 h.

c. SK-Hep1 cells were transiently transfected with Flag-PHGDH WT, Flag-PHGDH S371A or Flag-PHGDH S371D. Cells were then treated with 0.5 mM H_2O_2 and 20 μM CQ for 3 h. Flag-PHGDH proteins were immunoprecipitated and phosphorylation of S371 residue was detected.

d. SFB-PHGDH was pulled down from SK-Hep1 cells stably expressing SFB-PHGDH treated with 0.5 mM H_2O_2 and 20 μM CQ for 0 h, 3 h or 8 h.

e. SK-Hep1 cells stably expressing shNT or shHSC70 were transiently transfected with Flag-PHGDH WT or Flag-PHGDH S371D.

f. Kinase prediction based on S371 residue and surrounding residues by the kinase-library website, and the kinases were ranked as shown.

g. The proteins interacting with PHGDH were searched in Hitpredict database. And the protein kinases were ranked by interaction score.

h. The model of PHGDH structural domain. Sequence alignment of the PHGDH peptide (amino acid residues 364 to 379) across various species.

i. In vitro kinase assays were performed by mixing His-PHGDH WT with or without His-MAPK13 F324S. After the reaction, the sample was subjected to mass spectrometry to analyze the residues phosphorylated by MAPK13.

j. *Mapk13* mRNA levels in livers of WT and *Mapk13* KO mice were examined (n = 3).

k. SK-Hep1 cells were infected with a lentivirus expressing shNT or shMAPK13. PHGDH was immunoprecipitated from the cells treated with or without 0.5 mM H_2O_2 and 20 μM CQ for 3 h.

l. SK-Hep1 cells stably expressing shNT or shMAPK13 were treated with or without 0.5 mM

H_2O_2 for 24 h.

Immunoblots are representative of three independent experiments. Data are means ± SD. Each point represents an individual mouse. *P* values were determined by two-tailed Student's *t*-test.

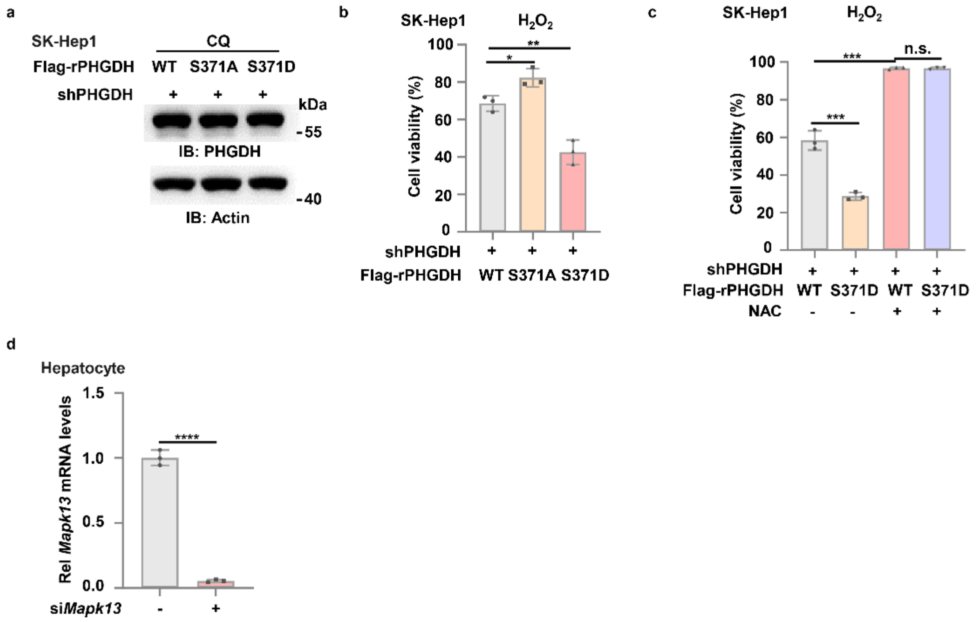

Supplementary Fig. S4 PHGDH pS371 promotes oxidative stress-induced cell death, related to Fig. 4.

a, b. SK-Hep1 cells stably expressing shPHGDH were infected with lentivirus expressing Flag-PHGDH WT, Flag-PHGDH S371A or Flag-PHGDH S371D. Cells then were treated with 20 μM CQ for 24h (**a**). Infected cells were treated with 0.5 mM H_2O_2 for 24 h. Cell viability was determined using trypan blue staining (**b**).

c. SK-Hep1 cells stably expressing shPHGDH were infected with lentivirus expressing Flag-rPHGDH WT or Flag-rPHGDH S371D respectively.We used0.5 mM H_2O_2 with or without 2.5 mM NAC to treat cells for 24 h. Cell viability was determined using trypan blue staining.

d. Hepatocytes isolated from *Phgdh*^{LKO} mice were transfected with siRNA of negative control (NC) or *Mapk13*. *Mapk13* mRNA levels of these cells were examined.

Immunoblots are representative of three independent experiments. Data represent the means ± SD of three independent experiments. *P* values were determined by two-tailed Student's *t*-test.

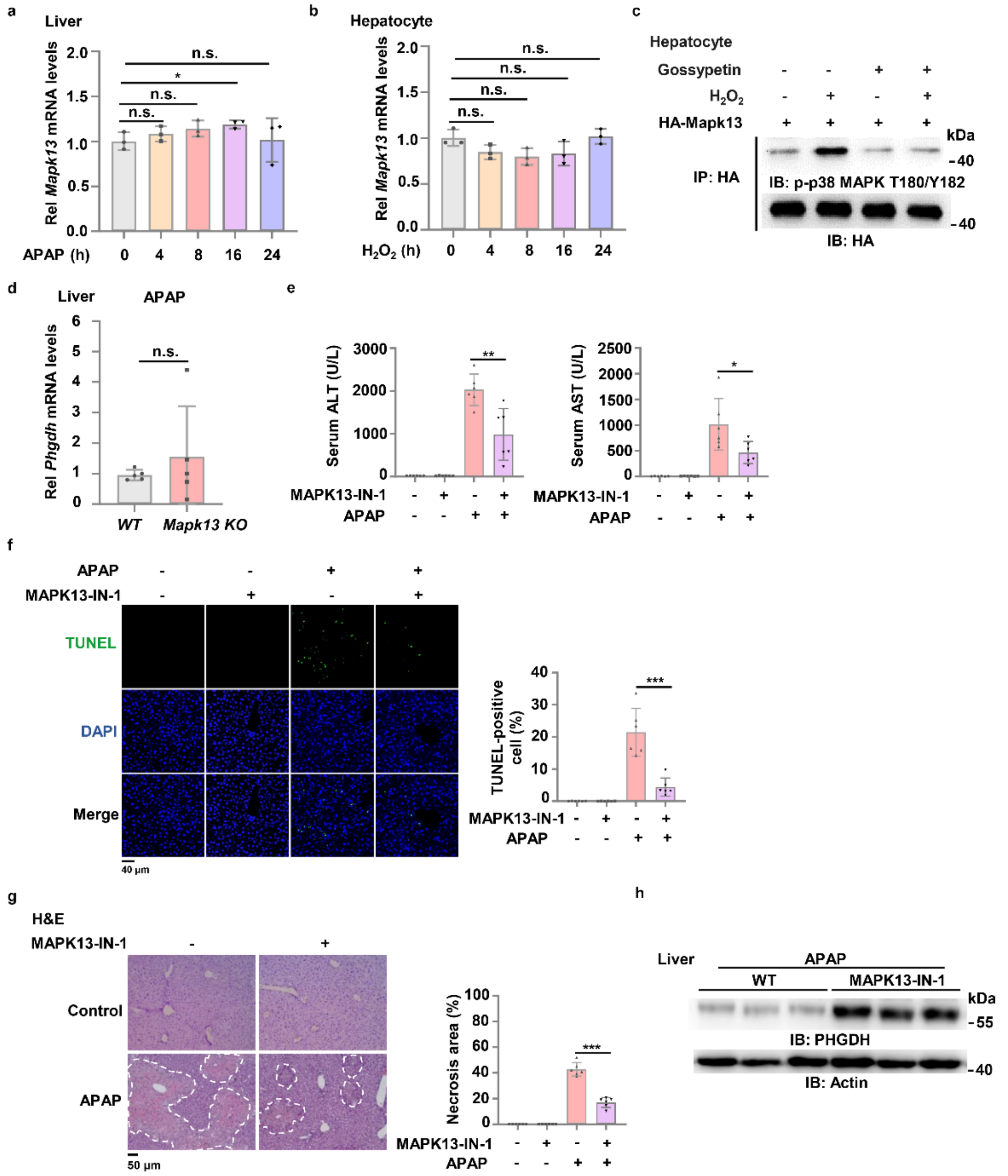

Supplementary Fig. S5 MAPK13 inhibition alleviates oxidative stress-associated liver injury, related to Fig. 5.

a. c57BL/6J mice were intraperitoneally injected with 300 mg/kg APAP. Livers were collected at 0 h, 4 h, 8 h, 16 h and 24 h after APAP treatment. *Mapk13* mRNA levels were examined (n = 3 in each time point).

b. Primary hepatocytes from c57BL/6J mice were treated with 0.5 mM H_2O_2 for 0 h, 4 h, 8 h, 16 h and 24 h. *Mapk13* mRNA levels were examined.

c. Primary hepatocytes isolated from c57BL/6J mice were transiently transfected with HA-Mapk13. Cells were then treated with 0.5 mM H_2O_2 and DMSO (control) or 50 μM Gossypetin for 4 h. HA-Mapk13 were immunoprecipitated and phosphorylation of Mapk13 was detected.

d. *WT* and *Mapk13 KO* mice were intraperitoneally injected with 300 mg/kg APAP. Mouse livers were collected at 16 h after treatment. *Phgdh* mRNA levels were examined (n = 5 per group).

e-h. c57BL/6J mice were intravenously injected with 5 mg/kg MAPK13-IN-1 solution or normal saline (control) 10 h after treated with normal saline (control) or 300 mg/kg APAP. After 24 h APAP treatment, serum ALT and AST levels in these mice were detected by kit (**e**). Positive liver cell in TUNEL-stained sections were quantified (**f**). Liver necrosis area in H&E-stained sections were circle and quantified (**g**). Representative images of H&E staining or TUNEL staining were shown on the left panel and statistical analysis were shown on the right panel (n = 5 per group). Immunoblotting analysis was performed to detect the protein levels of PHGDH in livers with or without MAPK13-IN-1 treatment after 24 h APAP treatment (**h**) (n = 3 per group).

Data represent means ± SD. Each point represents an individual mouse. P values were determined by two-tailed Student's t-test.

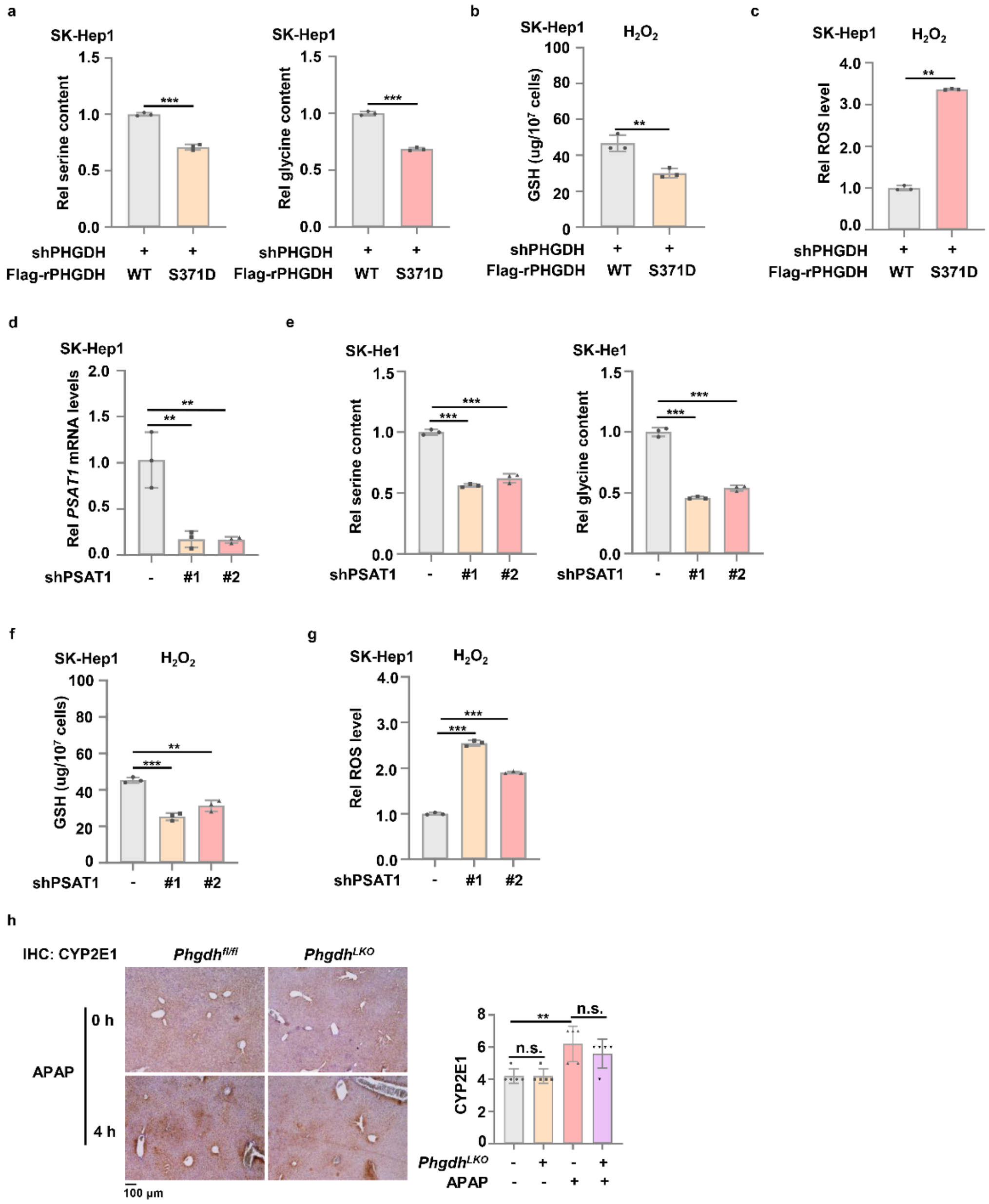

Supplementary Fig. S6 PHGDH pS371 inhibits GSH production, related to Fig. 6.

a. SK-Hep1 cells stably expressing shPHGDH were infected with lentivirus expressing Flag-PHGDH WT or Flag-PHGDH S371D. The serine and glycine contents in the cells were measured.

b, c. Cells in (**a**) were treated with 0.5 mM H_2O_2 for 12 h. GSH (**b**) and ROS (**c**) levels were measured.

d. SK-Hep1 cells were infected with lentivirus expressing shNT, shPSAT1 (#1) and shPSAT1 (#2). mRNA levels of *PSAT1* of these cells were examined.

e. The relative serine and glycine contents in the cells in (**d**) were measured by mass spectrometry.

f, g. Cells in (**d**) were treated with 0.5 mM H_2O_2 for 12 h. GSH (**f**) and ROS (**g**) levels were measured.

h. c57BL/6J mice were intraperitoneally injected with 300 mg/kg APAP. Mouse livers were collected at 0 h and 4 h after APAP treatment. IHC staining of liver section using anti-CYP2E1 antibody were performed. Representative images of IHC staining were presented on the left panel, and statistical analysis were shown on the right panel (n = 5).

Data represent the means ± SD of three independent experiments. P values were determined by two-tailed Student's t-test.

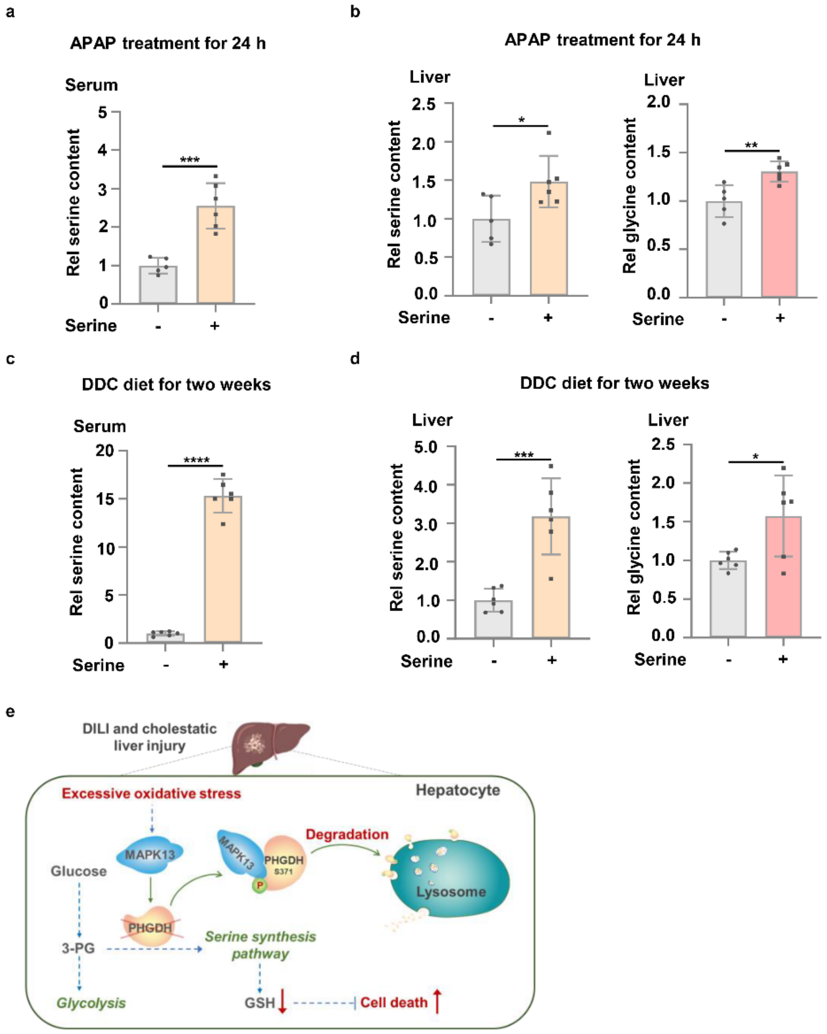

Supplementary Fig. S7 Serine supplementation promotes the elevation of serine and glycine levels in the liver, related to Fig. 7.

a, b. c57BL/6J mice were intraperitoneally injected with 300 mg/kg APAP for 24 h. And at 0 h and 12 h, mice were treated with 200 mg/kg serine solution or normal saline (control) by intragastric administration (i.g.). Then the relative content of serine in serum was detected by mass spectrometry (**a**). And relative content of serine and glycine in mouse liver were detected by mass spectrometry (**b**) (n = 5 in control group and n = 6 in serine supplementation group).

c, d. c57BL/6J mice were fed with 0.1 % DDC irradiated diet with or without 10 % serine for 2 weeks. The relative content of serine in the serum was detected by mass spectrometry (**c**). And relative content of serine and glycine in mouse liver were detected by mass spectrometry (**d**) (n = 6 per group).

e. Schematic illustration of the function and regulatory mechanism of PHGDH-related SSP in

oxidative stress-induced liver injury.

Data are means ± SD. Each point represents an individual mouse. *P* values were determined by two-tailed Student's *t*-test.

Supplementary Tables

Supplementary Table S1. Sequences of primers for real-time PCR

**Primer sequences for real-time PCR analysis**	**Sequence (5'-3')**
Phgdh-F	ATGGCCTTCGCAAATCTGC
Phgdh-R	GGAGTTCAGCTATCAGCTCCT
PHGDH-F	CTGCGGAAAGTGCTCATCAGT
PHGDH-R	TGGCAGAGCGAACAATAAGGC
Mapk13-F	GCATGTGCTTCAAGAGCAGAA
Mapk13-R	ATGGTGCATGTGCTTCAAGAG
PSAT1-F	GGCCAGTTCAGTGCTGTCC
PSAT1-R	GCTCCTGTCACCACATAGTCA
